# A systematic review and meta-analysis of quantitative pain outcomes in degenerative thoracic myelopathy

**DOI:** 10.3389/fnins.2026.1937393

**Published:** 2026-10-29

**Authors:** Zhikai Li, Philip Lee, Tanzil Rujeedawa, Hannah Louise Richmond, Natalia Jagodzinska, Georgios Toumbas, Benjamin M. Davies, Mark R. Kotter, Oliver D. Mowforth

**Affiliations:** 1 School of Clinical Medicine, University of Cambridge, Cambridge, United Kingdom; 2 Department of Clinical Neurosciences, University of Cambridge, Cambridge, United Kingdom

**Keywords:** degenerative thoracic myelopathy, ossification of the ligamentum flavum, ossification of the posterior longitudinal ligament, Oswestry disability index, thoracic disc herniation, visual analogue scale

## Abstract

**Introduction:**

Degenerative thoracic myelopathy (DTM) refers to symptomatic compression of the thoracic spinal cord secondary to degenerative pathology, including thoracic disc herniation (TDH), ossification of the ligamentum flavum (OLF) and ossification of the posterior longitudinal ligament (OPLL). Sensorimotor impairments and sphincter dysfunction often accompany pain, yet quantitative characterisation of pain and pain-related functional disability outcomes remains limited. We aimed to synthesise quantitative pain outcomes in DTM.

**Methods:**

The review was registered with PROSPERO (CRD420261297878) and conducted following PRISMA guidelines. MEDLINE and Embase were searched from inception until December 2025. Screening and data extraction were performed in duplicate and all risk-of-bias scoring was cross-checked. Findings were synthesised by random-effects meta-analyses for cohorts with sufficient data to estimate the standard deviation of change in pain. Cohorts not included in the meta-analysis were synthesised following the SWiM guidelines.

**Results:**

Twenty-eight studies comprising 1,225 patients within 34 cohorts met inclusion criteria. Every cohort improved after surgery (34/34 for the visual analogue scale (VAS); 10/10 for the Oswestry Disability Index (ODI)). In the 19 VAS cohorts that could be weighted (578 patients), the pooled reduction was 3.45 cm (95% CI 2.88–4.03; I^2^ = 94.5%), and in the nine ODI cohorts that could be weighted (145 patients) the pooled reduction was 29.4 points (20.9–37.8; I^2^ = 97.3%). The pooled VAS reduction was 4.06 cm in TDH and 3.34 cm in OLF cohorts. The 10 VAS cohorts that could not be weighted (436 patients) were synthesised descriptively, with a median of the reported mean reductions of 4.28 cm (IQR 3.85–4.63). Similar reductions were reported for discectomy with and without fusion. Only six studies reported axial pain separately from non-axial pain; consistent differentiation of pain subtypes and standardised reporting of pain measures are needed to strengthen the evidence base.

**Conclusion:**

Surgery for DTM appears to be associated with reductions in pain and pain-related functional disability, although substantial heterogeneity and the uncontrolled design of studies mean that certainty of the evidence is currently very low.

**Systematic review registration:**

PROSPERO, CRD420261297878.

## Introduction

Degenerative thoracic myelopathy (DTM) describes symptomatic thoracic spinal cord compression secondary to degenerative pathology, including thoracic disc herniation (TDH), ossification of the ligamentum flavum (OLF), and ossification of the posterior longitudinal ligament (OPLL) (Rujeedawa et al., 2024). Although conceptually related to degenerative cervical myelopathy (DCM), DTM is less common (Rujeedawa et al., 2024; Ando et al., 2019). As a result, DTM is relatively under-represented in the literature, with fewer studies and no consensus on optimal management (Rujeedawa et al., 2024; Bouthors et al., 2019; el-Kalliny et al., 1991; Court et al., 2018). Pain is a common presenting symptom and its nature varies, including axial and neuropathic subtypes (Rujeedawa et al., 2024). In addition, false localising patterns are common, complicating diagnosis and interpretation of outcomes (Rujeedawa et al., 2024).

Despite its clinical importance, pain in DTM is poorly characterised. Studies differ substantially in how pain is defined and measured, with inconsistent use of tools such as the visual analogue scale (VAS) and Oswestry Disability Index (ODI) (Bouthors et al., 2019; Fairbank & Pynsent, 2000). Many articles do not specify the type of pain assessed, and few report measures of dispersion or stratified outcomes to allow meaningful comparison across pathologies or surgical approaches (Bouthors et al., 2019; el-Kalliny et al., 1991; Court et al., 2018). This heterogeneity reflects both the relatively low prevalence of DTM and the predominance of retrospective single-arm cohorts, which often prioritise neurological or radiological outcomes over pain reporting. Consequently, the true burden of pain in DTM, and the extent to which surgery alleviates it, remains unclear.

Given these limitations, there is a clear need to synthesise the available evidence on pain outcomes in DTM. Improved understanding of how pain is reported, how it changes following surgery, and how outcomes vary between underlying aetiologies is essential for guiding clinical decision-making and informing future research. This review therefore aims to synthesise current evidence on pain outcomes following surgical management of DTM, highlighting inconsistencies in reporting, and identifying key knowledge gaps that must be addressed to improve the quality and comparability of future studies.

## Methods

### Study design

A systematic review was conducted in accordance with the Preferred Reporting Items for Systematic Reviews and Meta-Analyses (PRISMA) guidelines (Supplementary Table 1). The review protocol was registered on PROSPERO (CRD420261297878). The search strategy was developed iteratively using a combination of controlled vocabulary and free-text terms, with input from an experienced medical librarian. To assess search sensitivity, the final strategy was validated against a predefined set of 10 studies, with additional terms being incorporated until all key articles were consistently retrieved.

### Data sources and search strategies

A systematic literature search of Embase and MEDLINE using the Ovid platform (Ovid Technologies, New York, USA) was performed from database inception to December 12, 2025 (Supplementary Tables 2, 3). The search strategy was designed to maximise sensitivity by combining controlled vocabulary (e.g., MeSH terms) and free-text keywords relating to the thoracic spine, spinal cord compression and myelopathy, and degenerative spinal pathology including intervertebral disc disease and ligamentous pathology. Eligibility based on quantitative pain reporting and of surgical management was applied at title and abstract and full-text screening. No restrictions were placed on study design, publication type, or language at the search stage to ensure comprehensive retrieval. All records retrieved from both databases were exported into Rayyan (Rayyan Systems Inc., Cambridge, USA; Version 1.7.5) and subsequently into the reference manager Zotero (Corporation for Digital Scholarship, Fairfax, VA, USA; Version 9.0.4), where duplicates were removed using automated detection followed by manual verification prior to screening. In addition to database searching, reference lists of all included studies and relevant reviews were hand searched to identify additional eligible articles. Grey literature sources were also searched, and we considered any available unpublished work identified through expert knowledge. These supplementary searches were undertaken to minimise the risk of publication bias and ensure that articles not indexed in major databases were not overlooked.

### Study selection

Primary studies reporting quantitative assessment of pain outcomes, such as through the visual analogue scale (VAS) or Oswestry Disability Index (ODI), and any associated determinants including surgical intervention, were included. Four reviewers (PL, TR, ZL and HR) independently screened titles and abstracts in a blinded manner using Rayyan. Articles were assessed for eligibility using predefined inclusion and exclusion criteria (Table 1). An initial pilot screen of the first 100 articles was undertaken to ensure consistency between reviewers. Following unblinding, disagreements were resolved through discussion until consensus was reached. The same reviewers (PL, TR, ZL and HR) then performed duplicate full-text screening independently using the same eligibility criteria, followed by conflict resolution. Full texts were sought through institutional subscriptions and interlibrary request.

**Table 1:** Inclusion and exclusion criteria.

Inclusion	Exclusion
Adults with DTM Use of quantitative pain measurement tools Reported pain outcomes	Non-adult (<18 years) subjects Non-human subjects Systematic review or meta-analysis Editorial, case report, opinion article, letter, conference abstract, correction

### Data extraction

Four reviewers (PL, TR, ZL and HR) conducted data extraction, with data recorded in a standardised Excel spreadsheet (Microsoft, California, United States; Version 16.109.1). For each included study, we extracted mean VAS scores and/or ODI values before and after surgery, the mean duration of follow-up, the type of pain assessed (axial, radicular neuropathic, non-radicular neuropathic, or unspecified), the intervention performed, the primary pathology, sample size, and any reported *p*-values. For the meta-analysis we also extracted the standard deviation of the pre-operative score, the standard deviation of the score at final follow-up, the standard deviation of the change in pain score, and patient-level scores where these were tabulated (Supplementary Table 8). Where studies used VAS, this was taken to represent pain intensity on a 0–10 scale unless otherwise stated; ODI was interpreted as a percentage disability score, with higher values indicating greater pain-related functional impairment. Additional variables such as study design, demographic characteristics, and definitions of pain outcomes were also recorded, where available.

All extracted data were cross-checked for accuracy by a second reviewer. Any disagreements between reviewers were resolved through discussion and, if necessary, consultation with a third reviewer. Extracted data are summarised in Supplementary Tables 4, 5. All descriptive analyses were conducted using Excel.

### Data analysis and synthesis

Data handling and descriptive synthesis were performed using Microsoft Excel. All figures were drawn in Python 3.11 using Matplotlib. The included studies were heterogeneous in design, outcome measures and reporting formats, and fewer than half reported the standard deviation of the change in VAS or ODI, thus the analysis proceeded in two parts. Cohorts for which the standard deviation of the change could be estimated were pooled by random-effects meta-analysis. Cohorts not included in the meta-analysis were synthesised descriptively following the Synthesis Without Meta-analysis (SWiM) reporting guidelines (Supplementary Table 7) (Campbell et al., 2020). No cohort contributes both to a pooled estimate and to the descriptive synthesis of the same outcome.

The synthesis focused on pain outcomes following surgical management of DTM. Studies were grouped by aetiology (TDH, OLF, OPLL, mixed), surgical approach (discectomy with vs. without fusion) and types of pain (axial vs. non-axial). VAS is a measure of pain intensity. The ODI measures pain-related disability: the first of its 10 sections records pain intensity and the remaining nine record the extent to which pain limits a specific activity, therefore the total score reflects the functional burden of pain rather than pain intensity alone.

Where a study reported more than one aetiological group, surgical approach or type of pain, each was retained as a separate cohort, giving 34 VAS cohorts from 24 studies and 10 ODI cohorts from eight studies. Because several studies reported separate scores for more than one type of pain in the same patients, the sum of cohort sizes exceeds the number of individual patients; these sums are therefore reported as patient-observations rather than as patient counts.

Included studies reported mean values for VAS pain or ODI pre-operatively and at final follow up, from which mean change in VAS pain or ODI was derived. For the studies synthesised without meta-analysis, we report the distribution of these mean study-level values (median, IQR and range) and the direction of effect, following the SWiM reporting guidelines. Medians were chosen because each group contained few estimates and the median is less sensitive to outliers than the mean. We also report whether within-study changes were reported to be statistically significant. The standardised metric for synthesis was the mean change from baseline to final follow-up, calculated within each cohort as the difference between the mean pre-operative score and the mean score at final follow-up. Every reduction reported is therefore the median, interquartile range and range of these study-level mean changes, with quartiles obtained by linear interpolation. Because the median is not a linear operator, the median of the change is not the difference between the median pre-operative value and the median value at final follow-up; the median of the within-study change is the value reported throughout as the reduction.

### Meta-analysis

Meta-analyses of the change in pain score before and after surgery were performed in Python 3.11 (Python Software Foundation, Beaverton, USA) using NumPy and SciPy.

For 24 of the 34 VAS cohorts the standard deviation of the change could be extracted or imputed. Five of these cohorts, from four studies, represented a second pain domain measured in the same patients. For these studies, only the axial/back pain cohort entered the meta-analysis, such that no patient contributed twice. Data for non-axial pain were included in the SWiM analysis of axial vs. non-axial pain. Therefore, 19 cohorts from 16 studies, with a total of 578 patients were included in the VAS meta-analysis.

For 9/10 ODI cohorts, representing a total of 145 patients from seven studies, standard deviation could be extracted or imputed (Li et al., 2013; Wentao et al., 2015; Lin et al., 2021; Siller et al., 2020; Li et al., 2021; Wu et al., 2021; Hwang et al., 2022). The tenth study reported a range alone (Nacar et al., 2013). For one cohort, the standard deviation of the change was reported directly (Wu et al., 2021) and for the remaining eight it was imputed, using the same expression and correlation coefficient as for VAS. The correlation coefficient could be recovered in one study (Hwang et al., 2022), which gave 0.58 and 0.57 in its two cohorts.

Cohorts were pooled using the DerSimonian & Laird (1986) random-effects model, with the mean reduction in VAS from baseline to final follow-up as the effect measure. Where a study did not report the standard deviation of the change it was imputed as √(*a*^2^ + *b*^2^ − 2*rab*), where *a* is the standard deviation of the pre-operative score, *b* is the standard deviation of the score at final follow-up and *r* is the correlation coefficient between the two within patient scores, as described in the Cochrane Handbook for Systematic Reviews of Interventions (Higgins et al., 2024). Studies reporting all three standard deviations, or patient-level scores, allowed *r* to be derived by rearranging the same formula, and *r* was set to the median of the derived values. The primary analysis used *r* = 0.50, with *r* = 0.25 and *r* = 0.75 as sensitivity analyses. The same model, the same imputation and the same value of *r* were applied to the ODI cohorts.

For VAS, the standard deviation of the change was reported for four cohorts from three studies (Wu et al., 2021; Zhang et al., 2022; Baram et al., 2025). For one cohort, patient-level scores were reported, from which the standard deviation of the change was calculated (Lisitzky et al., 2022). For the remaining 14 cohorts from 12 studies, standard deviations of the pre-operative score and of the score at final follow-up were reported (Li et al., 2013; Wentao et al., 2015; Lin et al., 2021; Li et al., 2021; Hwang et al., 2022; Zhong et al., 2016; Yang et al., 2021; Krishnan et al., 2020; Xin et al., 2020; Deng et al., 2022; Kim et al., 2025; Ito S. et al., 2024), from which the standard deviation of the change was imputed. Across the eight cohorts for which standard deviations were reported or directly calculable, *r* was 0.65, 0.65, 0.66 and 0.67 in one study (Zhang et al., 2022), 0.26, 0.26 and 0.35 in another (Baram et al., 2025), and −0.16 in a third (Lisitzky et al., 2022). Their median was 0.50, which was used in the primary analysis. One study reported the change in pain and its standard deviation from a linear mixed model (Hwang et al., 2022); the correlations these imply, 0.27 to 0.57, were not used to set *r* but were consistent with the value chosen.

Heterogeneity was quantified by τ^2^, Cochran’s Q and I^2^, and a 95% prediction interval was calculated to describe the range within which the reduction of pain in a new cohort would be expected to fall.

Extensive sensitivity analyses included substitution of the second pain domain for the five studies reporting two domains, restriction of the analyses to the cohorts in which the standard deviation of the change was observed rather than imputed and omission of each cohort in turn in leave-one-out analysis. Small-study effects were examined by funnel plot and Egger’s test.

### Risk of bias and certainty of evidence assessments

Risk of bias was assessed using the Joanna Briggs Institute (JBI) critical appraisal tools matched to study design (Munn et al., 2020). Two reviewers (ZL and HR) conducted the assessment, with studies divided between them and all scoring cross-checked for consistency; any disagreements were resolved through discussion or consultation with a third reviewer. The six studies that included a concurrent comparator group were appraised with the JBI Critical Appraisal Checklist for Cohort Studies (Supplementary Table 6). The remaining 22 studies, of which 21 were single-arm cohorts without a comparator and one a contemporary series with a historical control group, were appraised using the JBI Critical Appraisal Checklist for Case Series (Munn et al., 2020) (Supplementary Table 6b). The JBI tools do not prescribe a specific scoring rule; we classified studies meeting at least 80% of the applicable items as low risk of bias, 50 to 79% as moderate and below 50% as high. Appraisals are presented in tabular form alongside a narrative synthesis.

The certainty of the evidence for each pooled outcome was rated using the Grading of Recommendations, Assessment, Development and Evaluations (GRADE) approach (Guyatt et al., 2011), across risk of bias, inconsistency, indirectness, imprecision and publication bias.

## Results

### Study selection

The search yielded 10,082 results (Figure 1). A total of 9,756 articles remained after de-duplication, with 97 following title and abstract screening. Most articles with pain in the title or abstract included no quantifiable pain measure and were excluded at full-text screening. Most of those that did include VAS reported a mean pre-operative score and mean post-operative scores at several time points. Reference list and grey-literature searching did not yield any additional eligible articles. Full-text screening yielded a total of 28 studies, 11 of which also reported ODI; eight of these could be attributed to a single aetiology and provide the 10 ODI cohorts analysed here.

**Figure 1:**
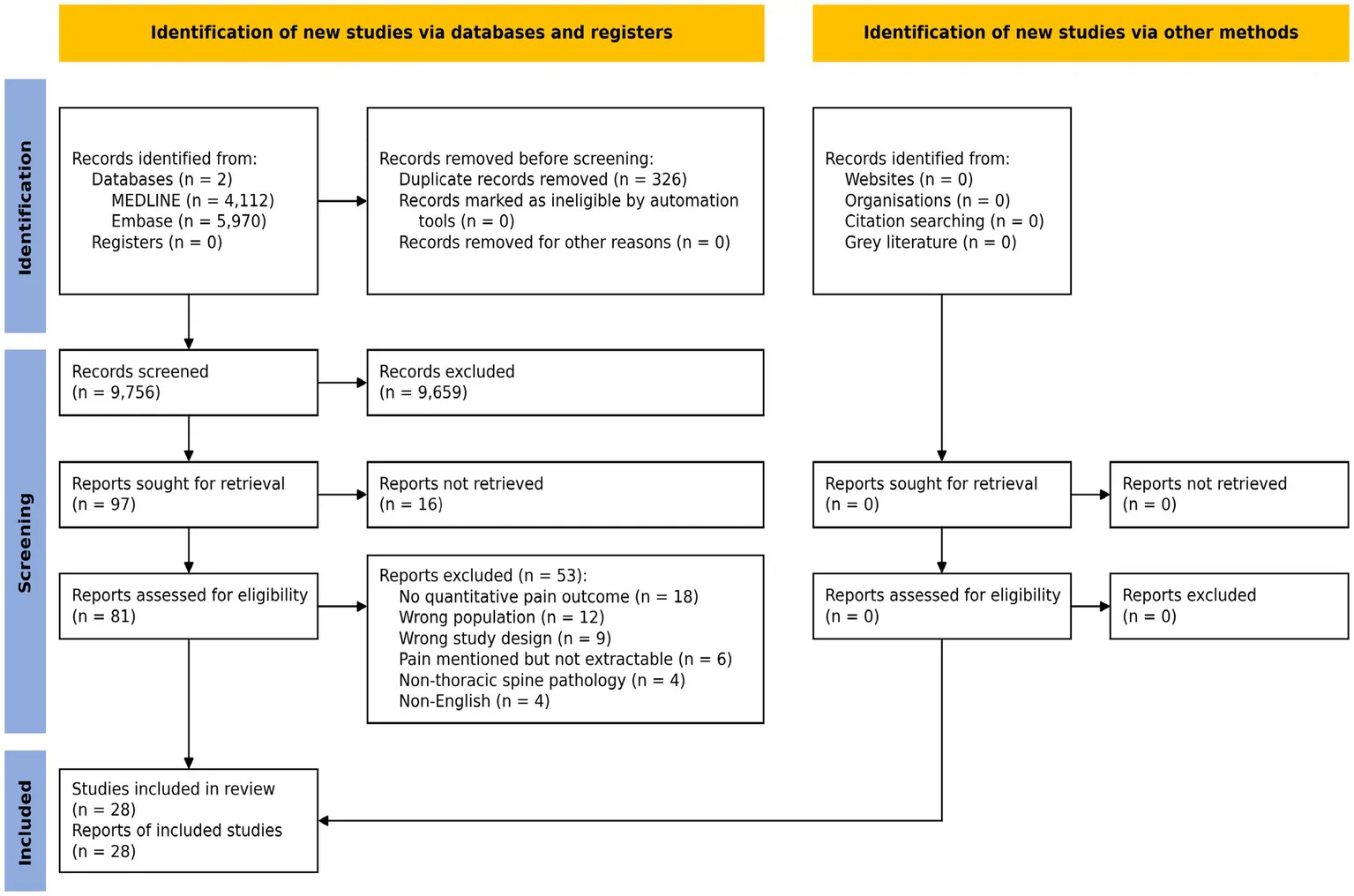
PRISMA 2020 flow diagram of study selection (Haddaway et al., 2022; Page et al., 2021). Of the 97 reports sought for retrieval, 16 could not be obtained despite institutional access and interlibrary requests, leaving 81 reports assessed for eligibility and 28 included in the final analysis.

### Sample characteristics

A total of 1,225 patients were included across the 28 studies (Li et al., 2013; Wentao et al., 2015; Lin et al., 2021; Siller et al., 2020; Li et al., 2021; Wu et al., 2021; Hwang et al., 2022; Nacar et al., 2013; Zhang et al., 2022; Baram et al., 2025; Lisitzky et al., 2022; Zhong et al., 2016; Yang et al., 2021; Krishnan et al., 2020; Xin et al., 2020; Deng et al., 2022; Kim et al., 2025; Ito S. et al., 2024; Bransford et al., 2010; Carr et al., 2017; Deviren et al., 2011; Uribe et al., 2012; Yen & Uribe, 2018; Quint et al., 2012; Soda et al., 2021; Ito Y. et al., 2024; Xu et al., 2025; Chen & Shi, 2024), with an unweighted mean age of 56.4 years. Across all included studies, the median sample size was 27.5 patients, with an interquartile range of 36 and a range of 157. Four were prospective cohort studies (4/28, 14.3%) and 24 were retrospective cohort studies (24/28, 85.7%). Six studies included a concurrent comparator group. Of the remaining 22 studies, one compared a contemporary series with a historical control group, and the remainder were single-arm series. A full summary of study characteristics is presented in (Supplementary Tables 4 and 5). Baseline risk factors and comorbidities were inconsistently reported and are therefore not presented here.

### Degenerative thoracic myelopathy Aetiologies

Thirteen studies reported TDH alone (13/28, 46.4%) (Wentao et al., 2015; Lin et al., 2021; Nacar et al., 2013; Baram et al., 2025; Lisitzky et al., 2022; Krishnan et al., 2020; Bransford et al., 2010; Carr et al., 2017; Deviren et al., 2011; Uribe et al., 2012; Yen & Uribe, 2018; Quint et al., 2012; Soda et al., 2021) and 11 reported ligament ossification alone (11/28, 39.3%) (Li et al., 2013; Li et al., 2021; Wu et al., 2021; Hwang et al., 2022; Zhang et al., 2022; Zhong et al., 2016; Yang et al., 2021; Xin et al., 2020; Deng et al., 2022; Kim et al., 2025; Ito S. et al., 2024), of which 10 were OLF and one was OPLL. The remaining four reported mixed aetiologies combining TDH, OLF and other causes such as OPLL (4/28, 14.3%) (Siller et al., 2020; Ito Y. et al., 2024; Xu et al., 2025; Chen & Shi, 2024). These four studies varied in the pathologies included and in how pain was recorded and were therefore not analysed by aetiology. Where reported, their pain and disability outcomes improved after surgery in the same direction as in the TDH and OLF studies.

### Meta-analysis of VAS pain

Pooled using a random-effects model, the mean reduction in VAS across the 19 cohorts was 3.45 cm (95% CI 2.88–4.03; Figure 2), with every cohort favouring surgery. Heterogeneity was substantial (I^2^ = 94.5%, τ^2^ = 1.49, Q(18) = 326.3, *p* < 0.001). The 95% prediction interval ranged from 0.80 to 6.10 cm, a magnitude of benefit ranging from less than the minimum clinically important difference (MCID) (Copay et al., 2008) to several times the pooled estimate. The mean reduction was 4.06 cm (2.74–5.38; I^2^ = 93.8%) across the five TDH cohorts and 3.34 cm (2.68–4.00; I^2^ = 94.6%) across the 13 OLF cohorts. Mean pain reduced by 1.90 cm (1.07–2.73) in the single OPLL cohort of 51 patients (Ito S. et al., 2024).

**Figure 2:**
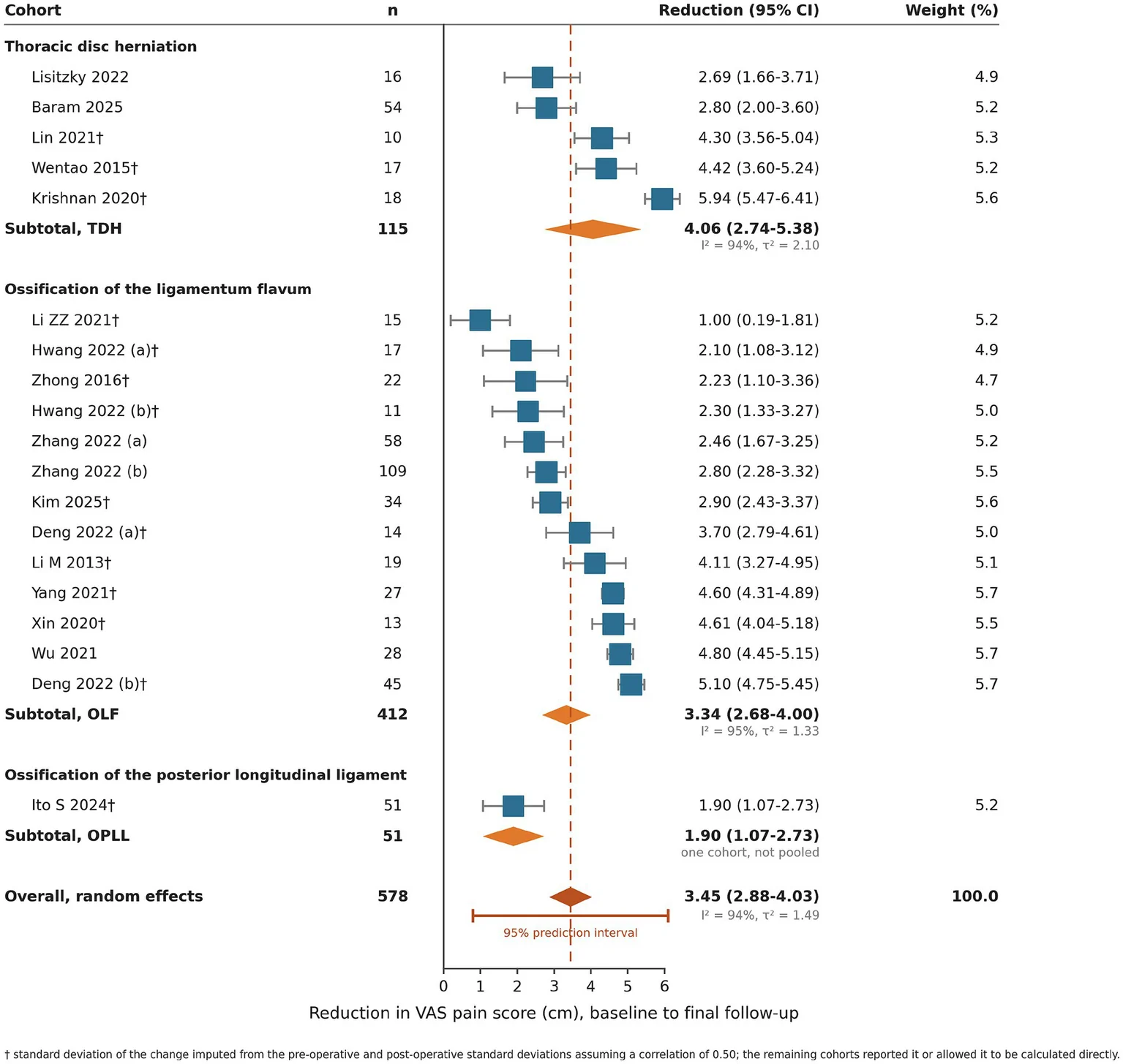
Random-effects meta-analysis of the reduction in VAS pain score from baseline to final follow-up, across the 19 cohorts in which the standard deviation of the change was reported or could be imputed, grouped by aetiology. Squares show each cohort’s mean reduction, sized by its weight in the random-effects model; horizontal lines represent the 95% confidence interval; diamonds show the pooled estimates, and the bar beneath the overall diamond shows the 95% prediction interval. Where a study contributed more than one cohort, these are labelled **(a)** and **(b)**.

The pooled estimate was insensitive to the assumed correlation used to impute the standard deviation of change in VAS pain, ranging from 3.46 cm (2.89–4.04) at *r* = 0.25 to 3.44 cm (2.86–4.02) at *r* = 0.75. Heterogeneity remained substantial regardless of the assumed correlation with I^2^ ranging from 92.9 to 96.5%. Restricting the analysis to the five cohorts in which the standard deviation of the change was observed rather than imputed gave a pooled estimate of 3.14 cm (2.00–4.28). Substituting the second pain domain from the five cohorts whose studies reported two domains in the same patients gave a pooled estimate of 3.64 cm (3.10–4.17). In leave-one-out analysis, omitting each cohort in turn moved the pooled estimate between 3.31 and 3.59 cm, indicating that no single cohort determined the overall result.

The cohorts were more widely scattered than sampling error alone would predict (Supplementary Figure 1), consistent with substantial heterogeneity. Egger’s regression intercept was significant (−6.69, *p* = 0.002). However, the direction of asymmetry was opposite to that expected for publication bias, with the least precise cohorts reporting smaller rather than larger reductions. Precision was driven more by the width of the post-operative distribution than by the sample size, and with I^2^ above 90%, tests of funnel plot asymmetry could not distinguish small study effects associated with heterogeneity from reporting bias. We therefore do not interpret this as evidence of publication bias.

### Meta-analysis of ODI

Pooled using a random-effects model, the mean reduction in ODI across nine cohorts was 29.4 points (95% CI 20.9–37.8; Figure 3), with every cohort favouring surgery. Heterogeneity was higher than for VAS (I^2^ = 97.3%, τ^2^ = 158.3, Q(8) = 294.6, *p* < 0.001). The 95% prediction interval ran from −2.1 to 60.8 points and includes no change. The assumed correlation again made almost no difference, the pooled estimate being 29.4 points (21.0–37.7) at *r* = 0.25 and 29.4 points (20.7–38.0) at *r* = 0.75. The mean reduction in ODI ranged between 27.8 and 31.9 points when omitting each cohort in leave-one-out analysis. Egger’s test gave no evidence of asymmetry (intercept −0.72, *p* = 0.89). Most of the heterogeneity arose from the two cohorts from a single study that began at a much lower baseline disability than the others and changed by approximately 10 points compared to 20–42 points in the remaining cohorts. Excluding those two cohorts, the pooled reduction across the remaining seven cohorts (117 patients) was 35.1 points (29.8–40.4), with I^2^ = 89.8%.

**Figure 3:**
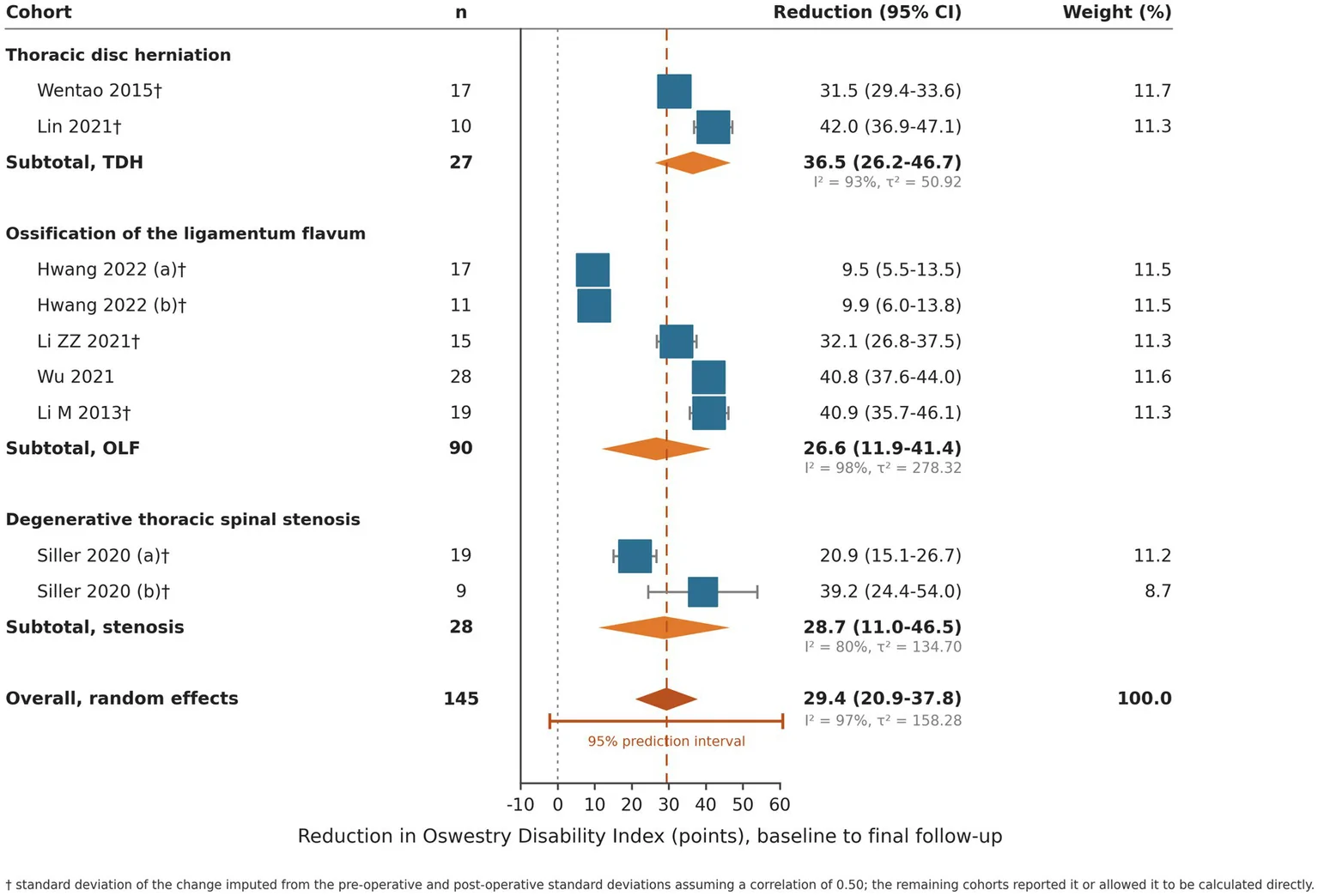
Random-effects meta-analysis of the reduction in ODI from baseline to final follow-up, across the nine cohorts in which the standard deviation of the change was reported or could be imputed. Squares show each cohort’s mean reduction, sized by its weight in the random-effects model, with horizontal lines giving the 95% confidence interval; diamonds show the pooled estimates, and the bar beneath the overall diamond shows the 95% prediction interval. Where a study contributed more than one cohort, these are labelled **(a)** and **(b)**.

### Studies not included in the meta-analysis

Ten of the 34 VAS cohorts, drawn from eight studies and 436 patients, reported neither the standard deviation of the change nor the standard deviations from which it could be imputed, and therefore could not be pooled. All 10 are thoracic disc herniation cohorts; every ossification of the ligamentum flavum cohort was included in the meta-analysis.

Every cohort improved in the direction favouring surgery (10/10). The median of the reported mean reductions in VAS was 4.28 cm (IQR 3.85–4.63; range 1.50–6.10). The median of the reported pre-operative means was 7.55 cm (IQR 6.21–8.10; range 4.70–9.00) and the median of the reported means at final follow-up was 3.00 cm (IQR 2.25–3.42; range 1.39–4.05; Figure 4).

**Figure 4:**
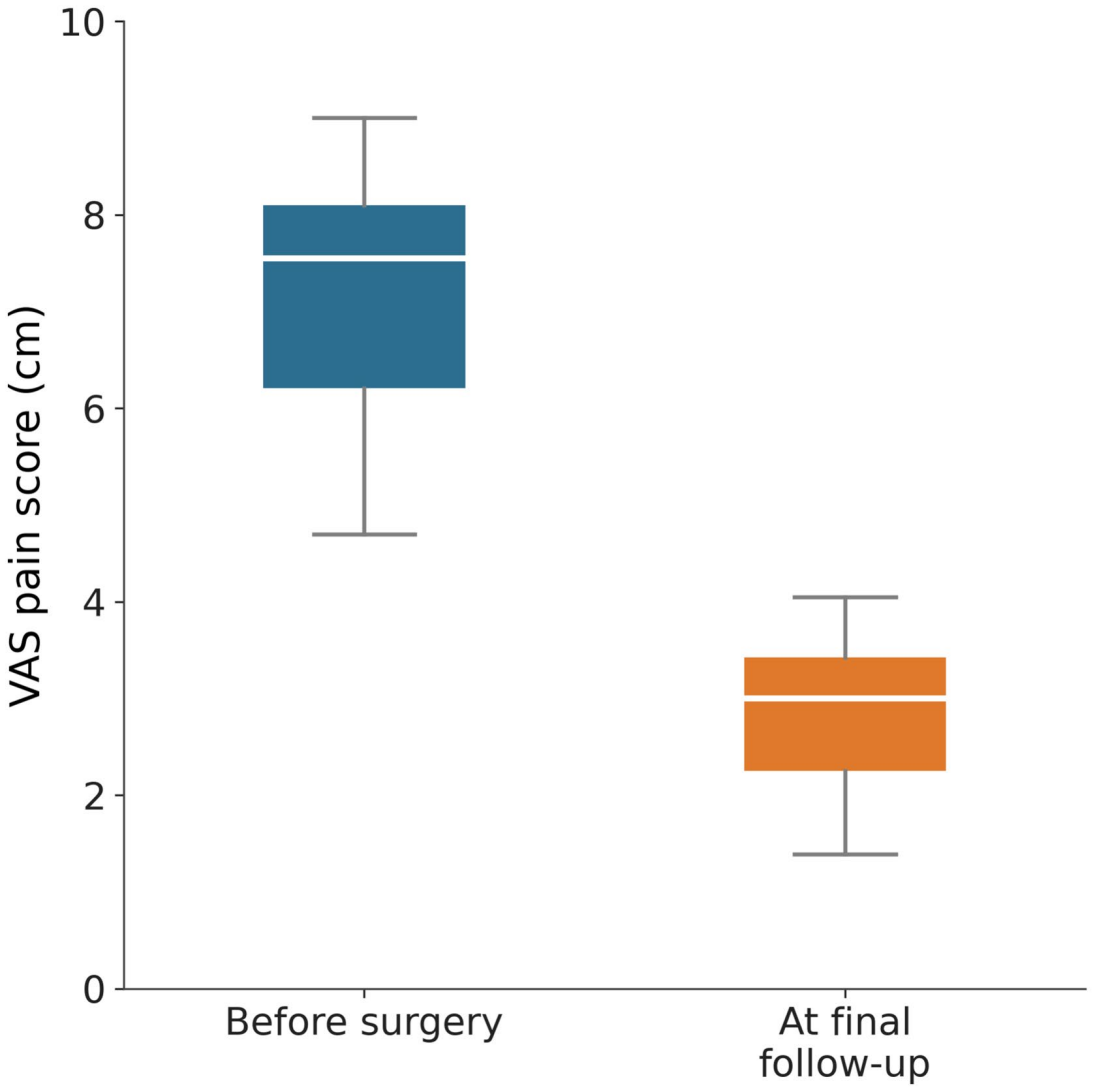
Distribution of mean VAS pain scores across the 10 cohorts that could not be included in the meta-analysis, before surgery and at final follow-up. Boxes show the median and interquartile range and whiskers show the full range.

### Discectomy with versus without fusion

The effect of adding interbody fusion to discectomy for TDH was also examined. Discectomy with fusion was performed for all patients in eight studies (Wentao et al., 2015; Nacar et al., 2013; Krishnan et al., 2020; Bransford et al., 2010; Carr et al., 2017; Deviren et al., 2011; Uribe et al., 2012; Yen & Uribe, 2018) contributing nine VAS cohorts, and was not performed in five studies (Lin et al., 2021; Lisitzky et al., 2022; Quint et al., 2012; Soda et al., 2021; Xu et al., 2025), contributing five VAS cohorts. One study performed discectomy with or without fusion, but did not stratify pain outcomes by procedure (Baram et al., 2025). Only two cohorts in each group reported the standard deviation of the change. This comparison could therefore not be pooled and is presented descriptively. Across the nine cohorts treated with fusion (217 patient-observations) the median of the reported mean reduction in VAS was 4.42 cm (IQR 4.26–5.50; range 3.80–6.10), and across the five cohorts treated without fusion (280 patient-observations) the median of the reported mean reduction in VAS was 3.61 cm (IQR 2.70–4.30; range 1.50–4.44) (Figure 5). Every cohort in both groups improved in the direction favouring surgery.

**Figure 5:**
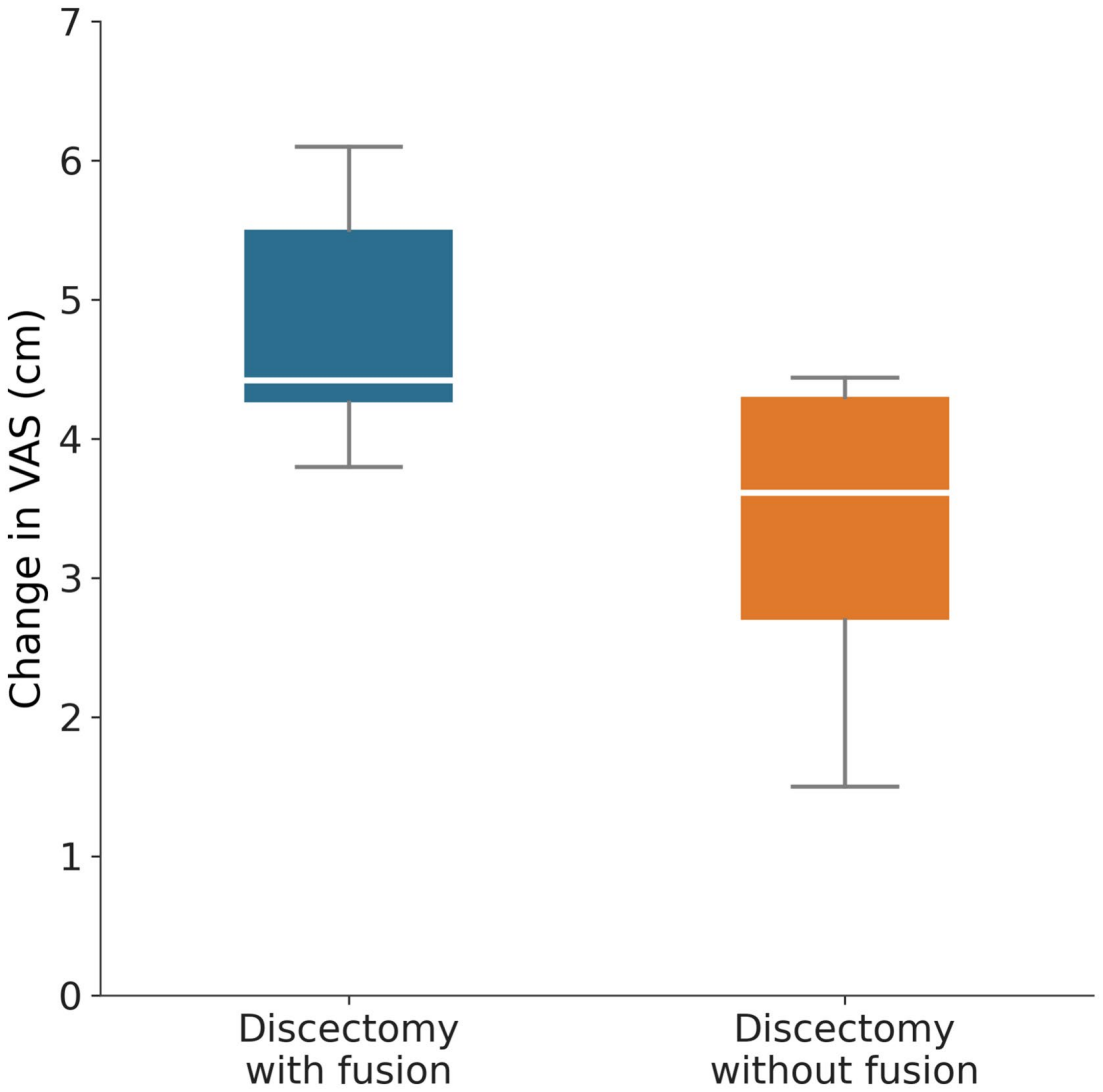
Distribution of the reported mean change in VAS pain score in TDH treated by discectomy with or without fusion. Boxes show the median and interquartile range across reported cohorts and whiskers show the full range.

### Axial versus non-axial pain

Six of the 28 studies (21.4%) reported axial back pain separately from a second, non-axial domain, allowing the two to be compared in the same patients. Two recorded radicular pain in a thoracic dermatomal distribution and four recorded leg pain. Both are grouped together here as non-axial pain. The remaining studies reported either a single composite pain score or the proportion of patients presenting with each pain type, without stratifying the outcome data.

Within these six studies the median of the reported mean reductions in axial pain was 2.04 cm (IQR 1.23–2.72; range 0.70–4.30) and in non-axial pain 2.33 cm (IQR 1.28–4.38; range 0.60–5.50; Figure 6). Both domains improved in every study that reported them separately. Non-axial pain improved more than axial pain in three studies, with the opposite true in the other three studies. Baseline severity was similar; the median of the reported mean pre-operative scores was 5.56 cm for axial pain and 5.12 cm for non-axial pain.

**Figure 6:**
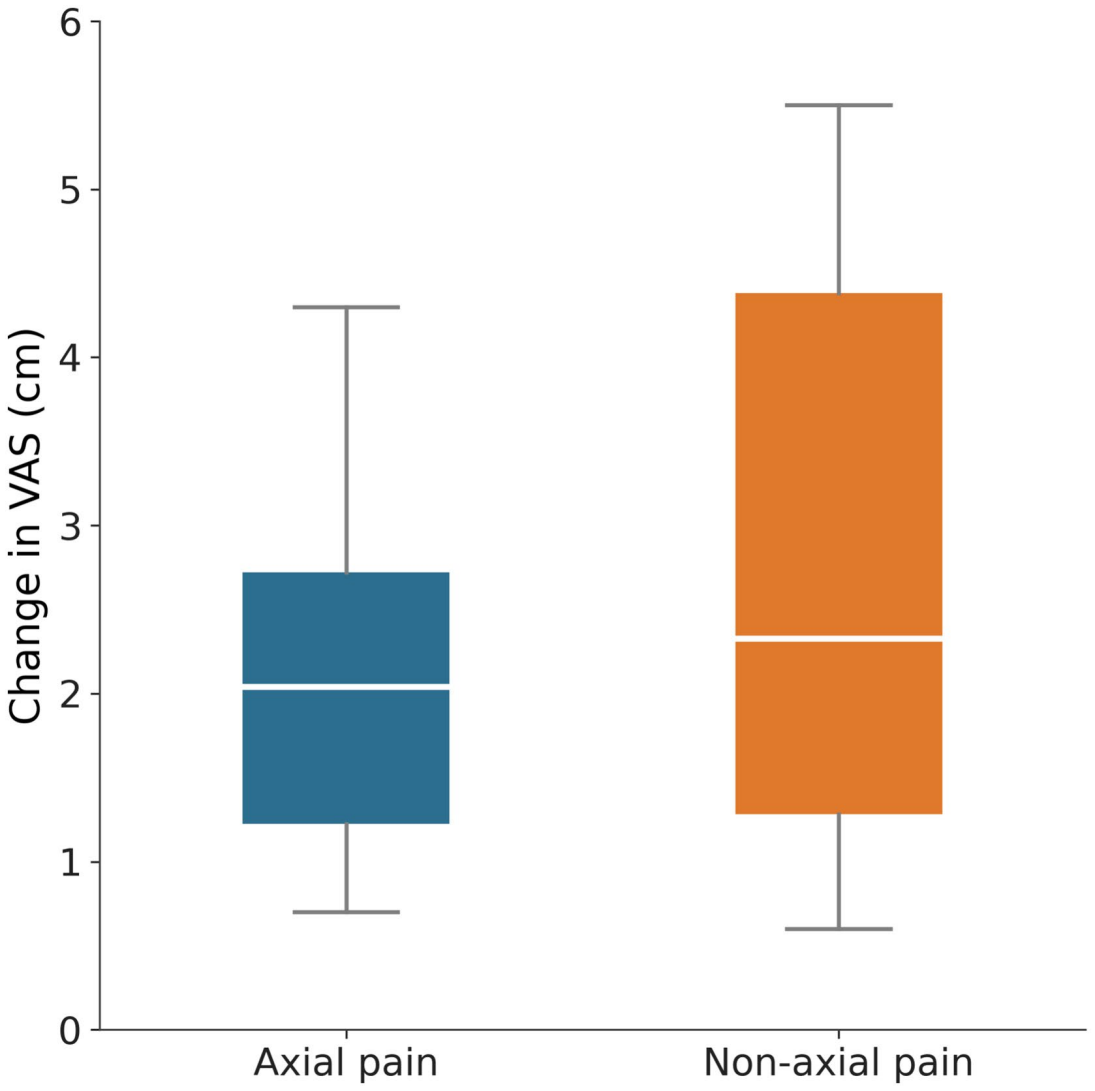
Distribution of the reported mean change in VAS pain score for axial and non-axial pain, across the six studies that reported the two domains separately. Boxes show the median and interquartile range and whiskers show the full range.

### Quality assessments

On assessment with the JBI Critical Appraisal Checklist for Case Series, 18 of the 22 studies without a comparator group were rated low risk of bias and four moderate risk (Supplementary Table 6b). All 22 studies measured and identified the condition in a standard, reliable way, and reported participant demographics, clinical information and the demographics of the presenting site. The items least often met concerned assembly of the series: consecutive inclusion was documented in 12 studies and complete inclusion in 12. The seven studies that did not achieve complete inclusion restricted eligibility to patients with complete records or a minimum duration of follow-up. Clear inclusion criteria were reported in 17 studies, and statistical analysis was judged appropriate in 17.

On assessment of the six studies with a comparator group using the JBI Critical Appraisal Checklist for Cohort Studies, one study was rated as low risk of bias and five were rated as moderate risk of bias (Supplementary Table 6a). All six studies drew both groups from the same institutional population, measured exposure and outcome in a valid and reliable way, reported the duration of follow-up and used an appropriate statistical analysis. The items least often met concerned confounding and follow-up. Only one study, which used propensity score matching, adjusted for confounding; the other five compared baseline characteristics between the groups without adjustment. Completeness of follow-up was unclear in four studies and not met in two.

Certainty of the evidence was very low for both pooled outcomes (Supplementary Table 9). For the reduction in both VAS and ODI, certainty began as low because every included study was observational, and was downgraded for risk of bias, as no study used blinded outcome assessment or adjusted for confounding, and for inconsistency given substantial heterogeneity. Neither outcome was downrated for indirectness, and neither showed evidence of publication bias.

## Discussion

### Summary of findings

Surgical management of degenerative thoracic myelopathy (DTM), particularly thoracic disc herniation (TDH) and ossification of the ligamentum flavum (OLF), appears to be consistently associated with significant pain alleviation. This review included 28 studies comprising over 1,200 patients, and every cohort improved in the direction favouring surgery. The consistency of this reduction across studies supports the role of surgical decompression in DTM. Conservative management has a limited evidence base, with analgesics and physiotherapy unable to address the underlying compressive pathology (Bouthors et al., 2019). This reinforces the clinical consensus that surgery is the definitive management for DTM (Rujeedawa et al., 2024), given its ability to address the causative pathology and improve both pain and neurological impairment (Lee et al., 2021).

The pooled cohorts had a mean reduction in VAS pain of 3.45 cm and in ODI of 29.4 points following surgery. Neither should be interpreted as the result to expect for an individual patient; heterogeneity was substantial and the ODI 95% prediction interval includes no change. The 10 VAS cohorts that could not be included in the meta-analysis improved without exception. Studies included in the meta-analysis and synthesis without meta-analysis were concordant on the direction of effect. Pain in DTM therefore appears to be a quantifiable target of treatment, consistent with prior scoping reviews that identified it as a central but under-quantified symptom (Rujeedawa et al., 2024).

For TDH, the pooled mean reduction in VAS was 4.06 cm. The median of the reported mean reductions was similar with and without fusion at 4.42 vs. 3.61 cm, respectively. Both approaches therefore appear to be associated with reduced pain, however unadjusted between-study data are insufficient to draw a firm conclusion on the relative efficacy of either procedure.

For OLF, the pooled mean reduction in VAS was 3.34 cm. The techniques used ranged from open laminectomy to full-endoscopic decompression. The available data therefore do not allow one approach to be compared with another without confounding. The choice of technique remains guided by clinical, anatomical and technical considerations rather than by any demonstrated difference in pain outcomes (Bouthors et al., 2019).

### Types of pain

Pain in DTM is heterogeneous and can be broadly categorised as axial or neuropathic (Tarulli, 2010). Axial pain may arise from structural degeneration, altered biomechanics, spinal instability, compensatory muscle strain and local inflammation (Ando et al., 2019; Tarulli, 2010). Patients typically describe it as a dull, aching discomfort that can be exacerbated by movement or prolonged standing.

Neuropathic pain may be radicular, radiating around the ribs or abdomen in a dermatomal distribution and described as sharp, burning or stabbing. Radicular pain results from nerve root entrapment and is frequently accompanied by paraesthesia (Buchowski et al., 2011). Neuropathic pain may also arise below the level of cord compression, without a radicular pattern, and manifest as diffuse leg pain with a burning or aching quality (Ando et al., 2019; Tarulli, 2010).

Included studies varied in their reporting of the type of pain. Some specified whether VAS scores referred to axial or neuropathic pain, while most reported overall pain scores without distinction. This hindered stratification of outcomes by pain type. Only six studies reported axial pain separately from non-axial pain, and among these the direction of the within-study difference was inconsistent, with non-axial pain improving more in three studies and axial pain more in the other three. Four of the six studies measured leg pain and two measured pain in a thoracic dermatomal distribution, which limits the comparison further. Nonetheless, both pain subtypes improved in every study that reported them separately. In the lumbar spine literature, patients with leg-dominant radicular symptoms often demonstrate greater postoperative improvement than those with axial pain alone (Divi et al., 2021; Kleinstück et al., 2009). However, six studies is too few to draw conclusions on whether one subtype responds preferentially to thoracic decompression and the absence of data stratified by pain type is an important evidence gap identified by this review. Differentiating pain types in future studies will be important to allow more nuanced conclusions.

### Clinical significance and relevance

Pain is often a primary symptom driving patients to seek medical attention for DTM, and as in DCM its alleviation is a key determinant of patient satisfaction (Mowforth et al., 2023; Davies et al., 2019). Demonstrating consistent and clinically significant reductions in VAS and ODI scores, which are both patient-reported outcome measures, provides strong evidence that surgery is effective not only in halting neurological decline but also in improving quality of life. However, better quality comparative evidence is needed on whether there is a difference in pain outcomes between surgical techniques. This may be particularly relevant in thoracic surgery, where surgical approaches are technically demanding, and complication risks can be higher than in cervical or lumbar surgery (Kato, 2020).

Reported changes in pain scores were also considered against published minimum clinically important difference (MCID) thresholds for spine surgery populations, which are approximately 1.5 to 2.0 cm for VAS and 10–12% for ODI (Copay et al., 2008). Twenty-nine of the 34 reported VAS cohorts (85.3%) exceeded the upper threshold of 2.0 cm and 31 (91.2%) met or exceeded 1.5 cm. The five cohorts falling below 2.0 cm had a median pre-operative VAS of 4.7 cm, which was below the median of the mean pre-operative scores across all cohorts (5.9 cm). This group included the lowest-scoring cohort in the review, with a pre-operative VAS of 1.4 cm, in which a large absolute change is not attainable. Seven of the 10 ODI cohorts (70%) exceeded a reduction of 12%. The reduction in every aetiology and surgical subgroup met or exceeded 1.5 cm, and every subgroup except the single thoracic OPLL cohort exceeded 2.0 cm. The improvements reported after surgery for DTM therefore appear clinically meaningful and not merely statistically detectable, with the caveat that these MCID values for pain are derived from lumbar and cervical populations and have not been validated in thoracic myelopathy.

However, whilst surgery for DTM appears to be associated with reduction in pain, the magnitude of improvement varied and high heterogeneity between studies led to a broad prediction interval for future studies. In addition, whilst pain may be reduced postoperatively, many patients experience ongoing pain of some degree. This highlights the need for clinicians to counsel patients realistically about expected outcomes.

### Limitations

The evidence on pain in DTM is currently from uncontrolled studies: 22 of the 28 studies were series without a comparator group, and none used blinded outcome assessment or adjusted for confounding. The direction of effect, that pain improves after surgery, is consistent across every reported cohort and is unlikely to be an artefact of any single study, but the magnitude of improvement and relative comparison between different aetiologies, surgical approaches and types of pain remains unclear. Rated with GRADE, the certainty of the evidence is very low for both pooled outcomes on account of the observational nature of studies and the substantial heterogeneity between them (Supplementary Table 9).

The risk of bias assessment items least often met were consecutive and complete inclusion; seven studies restricted eligibility to patients with complete records or a minimum duration of follow-up, a selection that may tend to favour patients who have recovered well following surgery. The evidence therefore does not support quantitative counselling of individual patients about the expected magnitude of improvement, nor a recommendation of any one surgical technique over another.

Standard deviations were also inconsistently reported. The meta-analysis therefore included 19/34 VAS cohorts and 578/1225 patients in the review, with an imputed standard deviation of the change in pain for 14 of these cohorts. The cohorts that could not be pooled were all TDH cohorts, with every OLF cohort pooled. The median year of publication was 2013 for the non-pooled cohorts compared to 2021 for the pooled cohorts. The pooled VAS estimate therefore reflects the more recently reported OLF literature more closely than older disc herniation series. In addition, the estimate is a pooled within-group change, not a treatment effect, and cannot separate the effect of surgery from natural history, regression to the mean or expectation. The ODI meta-analysis included 9/10 ODI cohorts and 145 patients, and rests on an imputed standard deviation of the change for eight cohorts. The ODI is a measure of pain-related disability rather than of pain intensity, and no included study reported its section-level scores.

The evidence base was also dominated by TDH and OLF with sparse data for other causes of thoracic myelopathy, such as OPLL (Rujeedawa et al., 2024; Ando et al., 2019). As a result, the generalisability of these findings to less common causes of DTM remains uncertain. Moreover, pain outcomes themselves were based on measures which capture intensity and functional disability but not pain quality or type (Seidenwurm, 2008), and were typically collected without blinded assessment. Furthermore, follow-up durations varied widely across studies, and although most studies reported sustained improvement, inconsistent reporting of follow-up procedures limits conclusions regarding long-term durability of pain relief. Finally, a total of 16 full texts could not be retrieved during screening, which increased the risk of selection bias.

### Future directions

Future research should prioritise standardised reporting of pain outcomes in DTM. Specifically, VAS and ODI scores should be reported with the standard deviation of the change, or with the pre-operative and post-operative standard deviations and the correlation between them, to enable studies to be pooled without imputation. Moreover, pain should be reported separately for axial and neuropathic pain, and within neuropathic pain for radicular and non-radicular pain, since these may respond differently to intervention. Furthermore, standardised time-points and longer duration of follow-up are needed to assess the durability of pain relief. In addition, comparative studies between surgical techniques should include pain as a primary outcome, not solely as a secondary measure. While randomised controlled trials may be challenging given the relatively low prevalence of DTM, registry-based studies could also provide higher-quality comparative evidence. International collaboration through networks such as AO Spine would facilitate adequately powered multi-centre studies.

## Conclusion

Surgical decompression is associated with reductions in pain and pain-related disability in DTM, although heterogeneity and the uncontrolled design of studies mean that the certainty of evidence is currently very low. Reductions in pain are consistently reported for both TDH and OLF cohorts, across multiple surgical approaches and for both axial and non-axial pain. Definitive comparisons between subgroups were not possible with currently available evidence. Future studies should standardise pain outcome measures, stratify by pain phenotype, and report the standard deviation of the change such that future meta-analysis need not rely on imputation.

## Data Availability

The original contributions presented in the study are included in the article/Supplementary material, further inquiries can be directed to the corresponding authors.
