# Supplementary material for: A systematic review and meta-analysis of quantitative pain outcomes in degenerative thoracic myelopathy
Source: Front Neurosci. 2026 Oct 29;20:1937393. doi: 10.3389/fnins.2026.1937393 (PMC13755334; doi:10.3389/fnins.2026.1937393)
Supplement: Supplementary file 1 [file Supplementary_file_1.docx]

**Supplementary Figures**

**Supplementary Figure 1.** Funnel plot of the 19 cohorts contributing to the meta-analysis of VAS. The vertical line marks the pooled estimate and the dashed lines enclose the region within which 95% of cohorts would be expected to fall in the absence of heterogeneity.

**Supplementary Table 1. PRISMA 2020 checklist.**

| **Section and Topic** | **Item #** | **Checklist item** | **Location where item is reported** |
| --- | --- | --- | --- |
| **TITLE** |  |  |  |
| Title | 1 | Identify the report as a systematic review. | Title page |
| **ABSTRACT** |  |  |  |
| Abstract | 2 | See the PRISMA 2020 for Abstracts checklist. | Abstract |
| **INTRODUCTION** |  |  |  |
| Rationale | 3 | Describe the rationale for the review in the context of existing knowledge. | Introduction |
| Objectives | 4 | Provide an explicit statement of the objective(s) or question(s) the review addresses. | Introduction, final paragraph |
| **METHODS** |  |  |  |
| Eligibility criteria | 5 | Specify the inclusion and exclusion criteria for the review and how studies were grouped for the syntheses. | Methods, Study Selection; Table 1 |
| Information sources | 6 | Specify all databases, registers, websites, organisations, reference lists and other sources searched or consulted to identify studies. Specify the date when each source was last searched or consulted. | Methods, Data Sources and Search Strategies |
| Search strategy | 7 | Present the full search strategies for all databases, registers and websites, including any filters and limits used. | Methods, Data Sources and Search Strategies; Supplementary Tables 2 and 3 |
| Selection process | 8 | Specify the methods used to decide whether a study met the inclusion criteria of the review, including how many reviewers screened each record and each report retrieved, whether they worked independently, and if applicable, details of automation tools used in the process. | Methods, Study Selection |
| Data collection process | 9 | Specify the methods used to collect data from reports, including how many reviewers collected data from each report, whether they worked independently, any processes for obtaining or confirming data from study investigators, and if applicable, details of automation tools used in the process. | Methods, Data Extraction |
| Data items | 10a | List and define all outcomes for which data were sought. Specify whether all results that were compatible with each outcome domain in each study were sought (e.g. for all measures, time points, analyses), and if not, the methods used to decide which results to collect. | Methods, Data Extraction |
|  | 10b | List and define all other variables for which data were sought (e.g. participant and intervention characteristics, funding sources). Describe any assumptions made about any missing or unclear information. | Methods, Data Extraction |
| Study risk of bias assessment | 11 | Specify the methods used to assess risk of bias in the included studies, including details of the tool(s) used, how many reviewers assessed each study and whether they worked independently, and if applicable, details of automation tools used in the process. | Methods, Risk of Bias and Certainty of Evidence Assessments |
| Effect measures | 12 | Specify for each outcome the effect measure(s) (e.g. risk ratio, mean difference) used in the synthesis or presentation of results. | Methods, Data Analysis and Synthesis; Methods, Meta-Analysis |
| Synthesis methods | 13a | Describe the processes used to decide which studies were eligible for each synthesis (e.g. tabulating the study intervention characteristics and comparing against the planned groups for each synthesis (item #5)). | Methods, Data Analysis and Synthesis |
|  | 13b | Describe any methods required to prepare the data for presentation or synthesis, such as handling of missing summary statistics, or data conversions. | Methods, Meta-Analysis |
|  | 13c | Describe any methods used to tabulate or visually display results of individual studies and syntheses. | Methods, Data Analysis and Synthesis; Supplementary Table 7 |
|  | 13d | Describe any methods used to synthesize results and provide a rationale for the choice(s). If meta-analysis was performed, describe the model(s), method(s) to identify the presence and extent of statistical heterogeneity, and software package(s) used. | Methods, Meta-Analysis |
|  | 13e | Describe any methods used to explore possible causes of heterogeneity among study results (e.g. subgroup analysis, meta-regression). | Methods, Meta-Analysis; subgroup analysis by aetiology and by surgical approach |
|  | 13f | Describe any sensitivity analyses conducted to assess robustness of the synthesized results. | Methods, Meta-Analysis; assumed correlation, observed standard deviations only, substitution of the second pain domain and leave-one-out analysis |
| Reporting bias assessment | 14 | Describe any methods used to assess risk of bias due to missing results in a synthesis (arising from reporting biases). | Methods, Meta-Analysis; funnel plot and Egger's test |
| Certainty assessment | 15 | Describe any methods used to assess certainty (or confidence) in the body of evidence for an outcome. | Methods, Risk of Bias and Certainty of Evidence Assessments |
| **RESULTS** |  |  |  |
| Study selection | 16a | Describe the results of the search and selection process, from the number of records identified in the search to the number of studies included in the review, ideally using a flow diagram. | Results, Study Selection; Figure 1 |
|  | 16b | Cite studies that might appear to meet the inclusion criteria, but which were excluded, and explain why they were excluded. | Results, Study Selection |
| Study characteristics | 17 | Cite each included study and present its characteristics. | Results, Sample Characteristics; Supplementary Tables 4 and 5 |
| Risk of bias in studies | 18 | Present assessments of risk of bias for each included study. | Results, Quality Assessments; Supplementary Tables 6a and 6b |
| Results of individual studies | 19 | For all outcomes, present, for each study: (a) summary statistics for each group (where appropriate) and (b) an effect estimate and its precision (e.g. confidence/credible interval), ideally using structured tables or plots. | Figures 2 and 3; Supplementary Tables 4, 5 and 8 |
| Results of syntheses | 20a | For each synthesis, briefly summarise the characteristics and risk of bias among contributing studies. | Results, Meta-Analysis of VAS Pain; Results, Meta-Analysis of ODI |
|  | 20b | Present results of all statistical syntheses conducted. If meta-analysis was done, present for each the summary estimate and its precision (e.g. confidence/credible interval) and measures of statistical heterogeneity. If comparing groups, describe the direction of the effect. | Results, Meta-Analysis of VAS Pain; Results, Meta-Analysis of ODI; Results, Studies Not Included in the Meta-Analysis |
|  | 20c | Present results of all investigations of possible causes of heterogeneity among study results. | Results, Meta-Analysis of VAS Pain; Results, Meta-Analysis of ODI |
|  | 20d | Present results of all sensitivity analyses conducted to assess the robustness of the synthesized results. | Results, Meta-Analysis of VAS Pain; Results, Meta-Analysis of ODI |
| Reporting biases | 21 | Present assessments of risk of bias due to missing results (arising from reporting biases) for each synthesis assessed. | Results, Meta-Analysis of VAS Pain; Supplementary Figure 1 |
| Certainty of evidence | 22 | Present assessments of certainty (or confidence) in the body of evidence for each outcome assessed. | Results, Quality Assessments; Supplementary Table 9 |
| **DISCUSSION** |  |  |  |
| Discussion | 23a | Provide a general interpretation of the results in the context of other evidence. | Discussion, Summary of Findings |
|  | 23b | Discuss any limitations of the evidence included in the review. | Discussion, Limitations |
|  | 23c | Discuss any limitations of the review processes used. | Discussion, Limitations |
|  | 23d | Discuss implications of the results for practice, policy, and future research. | Discussion, Clinical Significance and Relevance; Discussion, Future Directions |
| **OTHER INFORMATION** |  |  |  |
| Registration and protocol | 24a | Provide registration information for the review, including register name and registration number, or state that the review was not registered. | Abstract; Methods, Study Design |
|  | 24b | Indicate where the review protocol can be accessed, or state that a protocol was not prepared. | Methods, Study Design |
|  | 24c | Describe and explain any amendments to information provided at registration or in the protocol. | Not applicable; no amendments were made |
| Support | 25 | Describe sources of financial or non-financial support for the review, and the role of the funders or sponsors in the review. | Title page, Funding |
| Competing interests | 26 | Declare any competing interests of review authors. | Title page, Conflicts of interest |
| Availability of data, code and other materials | 27 | Report which of the following are publicly available and where they can be found: template data collection forms; data extracted from included studies; data used for all analyses; analytic code; any other materials used in the review. | Title page, Data Availability |

**Supplementary Table 2.** Search strategy for MEDLINE.

| **Ovid MEDLINE(R) and Epub Ahead of Print, In-Process, In-Data-Review & Other Non-Indexed Citations, Daily and Versions <1946 to December 12, 2025>** | | |
| --- | --- | --- |
| **#** | Searches | Number of Results |
| **1** | exp Thoracic Vertebrae/ or thora*.mp. | 391147 |
| **2** | exp spinal cord compression/ or exp spinal cord injuries/ or myelo*.mp. or spondylomyelo*.mp. or (spinal cord adj3 injur*).mp. or (Spinal Cord adj3 Compress*).mp. or exp Myelography/ | 483068 |
| **3** | degen*.mp. | 325518 |
| **4** | exp Spondylosis/ or exp Osteoarthritis/ or exp Zygapophyseal Joint/ or exp Spinal Stenosis/ or exp Joint Diseases/ or exp Spondylolisthesis/ or exp Joint Instability/ or exp Intervertebral Disc Displacement/ or exp Intervertebral Disc/ or exp Intervertebral Disc Degeneration/ or exp Spinal Osteophytosis/ or arthropathy.mp. or instability.mp. or facetjoint*.mp. or spondyl*.mp. or vertebral dis*.mp. or ((intervertebral dis* adj3 prolapse) or herniation or degeneration or protrusion or sequestration).mp. or osteo*.mp. or lax*.mp. or hypermobile*.mp. | 1494728 |
| **5** | exp Ehlers-Danlos Syndrome/ or exp Scheuermann Disease/ or Ehlers Danlos Syndrome.mp. or Scheuermann Disease.mp. | 6184 |
| **6** | exp Ligaments/ or exp Ossification of Posterior Longitudinal Ligament/ or exp Ligamentum Flavum/ or exp Longitudinal Ligaments/ or ((ligament adj2 calc*) or hypertroph* or ossif*).mp. or ligament* flavum.mp. or yellow ligament*.mp. or posterior longitudinal ligament*.mp. | 252599 |
| **7** | 3 or 4 or 5 or 6 | 1784880 |
| **8** | 1 and 2 and 7 | 4112 |

**Supplementary Table 3.** Search strategy for Embase.

| **Embase <1974 to 2025 December 12>** | | |
| --- | --- | --- |
| **#** | Searches | Number of Results |
| **1** | exp thoracic vertebra/ or exp thoracic spine/ or exp thoracic spinal cord/ or thora*.mp. | 796975 |
| **2** | exp Spinal cord compression/ or exp Spinal cord injury/ or myelopath*.mp. or spondylomyelo*.mp. or (Spinal Cord adj3 Compress*).mp. or (spinal cord adj3 injur*).mp. or exp Myelography/ | 124616 |
| **3** | exp degeneration/ or Degen*.mp. | 1290862 |
| **4** | exp Spondylosis/ or exp osteoarthritis/ or exp Spinal Osteoarthritis/ or exp Zygapophyseal Joint/ or exp Vertebral canal stenosis/ or exp Spondylosis deformans/ or exp joint degeneration/ or exp Arthropathy/ or exp joint instability/ or exp spondylolisthesis/ or exp intervertebral disk hernia/ or exp intervertebral disk/ or exp intervertebral disk degeneration/ or exp osteophyte/ or arthropathy.mp. or instability.mp. or facetjoint*.mp. or spondyl*.mp. or vertebral dis*.mp. or ((intervertebral dis* adj3 prolapse) or herniation or degeneration or protrusion or sequestration).mp. or osteo*.mp. or lax*.mp. or hypermobile*.mp. | 2142290 |
| **5** | exp Ehlers-Danlos Syndrome/ or exp Scheuermann Disease/ or exp Joint hypermobility/ or exp joint laxity/ or Ehlers-Danlos Syndrome.mp. or Scheuermann Disease.mp. | 14654 |
| **6** | exp ligament/ or exp Ligament calcinosis/ or exp ligamentum flavum/ or exp Posterior longitudinal ligament/ or ((ligament adj2 calc*) or hypertroph* or ossif*).mp. or ligament* flavum.mp. or yellow ligament*.mp. or posterior longitudinal ligament*.mp. | 423887 |
| **7** | 3 or 4 or 5 or 6 | 3197135 |
| **8** | 1 and 2 and 7 | 5970 |

**Supplementary Table 4. VAS data summary.** N/A denotes a *p*-value that was not reported by the source study. Where a study reported more than one cohort or more than one pain domain, each is shown on a separate row and carries its own sample size.

| **Study #** | **Cause of DTM** | **Intervention Category** | **Type of Pain (if specified)** | **No. of Patients** | **Mean Follow-Up Duration (Months)** | **Preoperative Average VAS (cm)** | **Final Follow-up Average VAS (cm)** | **Mean VAS Change** | **P-Value (VAS)** |
| --- | --- | --- | --- | --- | --- | --- | --- | --- | --- |
| Wentao et al., 2015^9^ | (TDH) Thoracic Disc Herniation | Laminectomy, Discectomy and Interbody Fusion | Back pain (100%); Radicular pain (76%) | 17 | 13.4 | 6.65 | 2.23 | 4.42 | *p* < 0.01 |
| Zhong et al., 2016^21^ | (OLF) Ossification of the Thoracic Ligamentum Flavum | Laminectomy (removal of laminae, OLF and medial half of facet joint) | Back and/or leg pain (50%) | 22 | 35.6 | 2.82 | 0.59 | 2.23 | *p* = 0.001 |
| Bransford et al., 2010^32^ | (TDH) Thoracic Disc Herniation | Discectomy and Interbody Fusion | Axial back pain | 14 | 12.2 | 5.9 | 2.1 | 3.8 | N/A |
| Yang et al., 2021^22^ | (OLF) Ossification of the Thoracic Ligamentum Flavum | Laminotomy | Leg pain | 27 | 14.3 | 5.9 | 1.3 | 4.6 | *p* < 0.001 |
| Zhang et al., 2022^18^ | (OLF) Ossification of the Thoracic Ligamentum Flavum (DISH-) | Laminectomy (only) and Laminectomy with Fusion | Overall | 109 | 36.7 | 3.45 | 0.65 | 2.8 | N/A |
|  | (OLF) Ossification of the Thoracic Ligamentum Flavum (DISH+) | Laminectomy (only) and Laminectomy with Fusion | (Back pain) | 58 | 36.7 | 3.37 | 0.91 | 2.46 | N/A |
| Carr et al., 2017^33^ | (TDH) Thoracic Disc Herniation | Discectomy and Interbody Fusion | Back pain (20%); Radicular pain (35%) | 51 | 14 | 8.31 | 4.05 | 4.26 | *p* < 0.01 |
| Deviren et al., 2011^34^ | (TDH) Thoracic Disc Herniation | Discectomy and Interbody Fusion | Back pain (92%); Leg pain (83%) | 12 | 28 | 9 | 2.9 | 6.1 | *p* < 0.05 |
| Uribe et al., 2012^35^ | (TDH) Thoracic Disc Herniation | Discectomy and Interbody Fusion | Back pain (77%) | 60 | 11 | 7.8 | 3.1 | 4.7 | N/A |
| Yen et al., 2018^36^ | (TDH) Thoracic Disc Herniation | Discectomy and Interbody Fusion | Axial back pain (22%) | 5 | 15.4 | 8.2 | 3.9 | 4.3 | N/A |
|  | (TDH) Thoracic Disc Herniation | Discectomy and Interbody Fusion | Radicular pain (30%) | 7 | 15.4 | 7.6 | 2.1 | 5.5 | N/A |
| Lin et al., 2021^10^ | (TDH) Thoracic Disc Herniation | Discectomy | Back pain; Radicular | 10 | 12 | 6.1 | 1.8 | 4.3 | *p* < 0.05 |
| Siller et al., 2020^11^ | Degenerative thoracic spinal stenosis | Hemi/Laminectomy | Pain (26%) | 19 | 35.1 | 7 | 2 | 5 | *p* = 0.038 |
|  | Degenerative thoracic spinal stenosis | Hemi/Laminectomy | Pain (11%) | 9 | 35.1 | 5 | 1 | 4 | *p* = 0.035 |
| Lisitzky et al., 2022^20^ | (TDH) Thoracic Disc Herniation | Discectomy | Axial back pain; Radiculopathy | 16 | 12 | 4.8 | 2.1 | 2.7 | N/A |
| Nacar et al., 2013^15^ | (TDH) Thoracic Disc Herniation | Discectomy and Interbody Fusion | Axial back pain (94%); Radiculopathy (94%); Leg pain (70%) | 33 | 18.2 | 7.5 | 3.5 | 4 | *p* < 0.05 |
| Li et al., 2013^8^ | (OLF) Ossification of the Thoracic Ligamentum Flavum | Laminectomy | Back pain (42%); Leg pain (11%) | 19 | 53.2 | 6.29 | 2.18 | 4.11 | *p* < 0.05 |
| Quint et al., 2012^37^ | (TDH) Thoracic Disc Herniation | Micro-Discectomy | Axial back pain only (10%); Radicular pain (52%); Mixed (37%) | 167 | 24 | 7.14 | 2.7 | 4.44 | *p* < 0.05 |
| Krishnan et al., 2020^23^ | Thoracic Disc Prolapse | Facetectomy, Laminectomy and Interbody Fusion | Back pain | 18 | 65.05 | 6.94 | 1 | 5.94 | *p* < 0.0001 |
| Xin et al., 2020^24^ | (OLF) Ossification of the Thoracic Ligamentum Flavum | Flavectomy | Back pain | 13 | 29.2 | 5.3 | 0.69 | 4.61 | *p* = 0.001 |
| Soda et al., 2021^38^ | (TDH) Thoracic Disc Herniation | Discectomy | Radicular pain (59%) | 49 | 46 | 4.7 | 3.2 | 1.5 | *p* = 0.0019 |
|  | (TDH) Thoracic Disc Herniation | Discectomy |  | 38 | 46 | 5 | 1.39 | 3.61 | *p* < 0.05 |
| Li et al., 2021^12^ | (OLF) Ossification of the Thoracic Ligamentum Flavum | En Bloc Resection/laminectomy | Back pain (100%) | 15 | 24 | 1.4 | 0.4 | 1 | *p* < 0.05 |
|  | (OLF) Ossification of the Thoracic Ligamentum Flavum | En Bloc Resection/laminectomy | Radicular (100%) |  | 24 | 2.8 | 0.27 | 2.53 | *p* < 0.05 |
| Deng et al., 2022^25^ | (OLF) Ossification of the Thoracic Ligamentum Flavum | Laminectomy | Leg pain (100%) | 14 | 12 | 4.5 | 0.8 | 3.7 | *p* < 0.001 |
|  | (OLF) Ossification of the Thoracic Ligamentum Flavum | Laminectomy | Leg pain (100%) | 45 | 12 | 6.6 | 1.5 | 5.1 | *p* < 0.001 |
| Wu et al., 2021^13^ | (OLF) Ossification of the Thoracic Ligamentum Flavum | Laminotomy, Bilateral Decompression using One Block Resection | Back pain | 28 | 10 | 6.2 | 1.4 | 4.8 | *p* < 0.05 |
| Hwang et al., 2022^14^ | (OLF) Ossification of the Thoracic Ligamentum Flavum | Laminotomy without fusion | Back pain | 17 | 24 | 5.2 | 3.1 | 2.1 | *p* = 0.01 |
|  | (OLF) Ossification of the Thoracic Ligamentum Flavum | Laminotomy with fusion | Back pain | 11 | 24 | 6.5 | 4.2 | 2.3 | *p* < 0.01 |
|  | (OLF) Ossification of the Thoracic Ligamentum Flavum | Laminotomy without fusion | Leg pain |  | 24 | 5.8 | 4.5 | 1.3 | *p* = 0.01 |
|  | (OLF) Ossification of the Thoracic Ligamentum Flavum | Laminotomy with fusion | Leg pain |  | 24 | 6.9 | 3.5 | 3.4 | *p* < 0.01 |
| Kim et al., 2025^26^ | (OLF) Ossification of the Thoracic Ligamentum Flavum | Laminectomy | Back pain | 34 | 12.7 | 5.8 | 2.9 | 2.9 | N/A |
|  | (OLF) Ossification of the Thoracic Ligamentum Flavum | Laminectomy | Leg pain |  | 12.7 | 7.4 | 2.4 | 5 | N/A |
| Ito Y et al., 2024^39^ | Mixed (thoracic spondylotic myelopathy, OPLL, OLTF, and disk herniation) | Decompression with or without fusion | Low back pain | 90 | 12 | 3.6 | 2.9 | 0.7 | *p* < 0.001 |
|  |  |  | Leg pain |  | 12 | 3.8 | 3.2 | **0.6** | *p* < 0.001 |
|  |  |  | Plantar pain |  | 12 | 2.5 | 2.1 | **0.4** | *p* < 0.001 |
| Baram et al., 2025^19^ | (TDH) Thoracic Disc Herniation | Discectomy +/- laminectomy +/- fusion | Anterior approach | 34 | 70 | 2.7 | 0.6 | **2.1** | N/A |
|  |  |  | Posterior approach | 20 | 70 | 5.4 | 1.3 | **4.1** | N/A |
|  |  |  | Overall | 54 | 70 | 3.7 | 0.9 | **2.8** | N/A |
| Ito S et al., 2024^27^ | (OPLL) Ossification of the Posterior Longitudinal Ligament | Laminectomy with fusion | Back pain | 51 | >120 | 5.4 | 3.5 | 1.9 | *p* < 0.001 |
|  | (OPLL) Ossification of the Posterior Longitudinal Ligament | Laminectomy with fusion | Leg pain | 51 | >120 | 4 | 3 | 1 | *p* < 0.001 |
| Chen et al., 2024^41^ | Mixed (OPLL, posterior vertebral osteophytes, hyperplasia of the facet joints, or OLTF) | Laminectomy with fusion | Overall | 38 | 24.5 | 5.05 | 1.66 | 3.39 | *p* < 0.001 |
|  | Mixed (OPLL, posterior vertebral osteophytes, hyperplasia of the facet joints, or OLTF) | Conservative | Overall | 26 | 24.5 | 4.96 | 4.58 | 0.38 | *p* = 0.086 |

**Supplementary Table 5. ODI data summary.** NR denotes a *p*-value that was not reported by the source study; a dash denotes an outcome that was not reported. The ODI summary reported in the Results draws on the eight studies whose ODI data could be attributed to thoracic disc herniation, ossification of the ligamentum flavum or degenerative thoracic stenosis; the three studies whose ODI data combined OPLL with other aetiologies could not be assigned to a single aetiology and are excluded from that summary.

| **Study #** | **Cause of DTM** | **Approach** | **Intervention Category** | **Intervention** | **Type of Pain (if specified)** | **No. of Patients** | **Mean Follow-Up Duration (Months)** | **Preoperative Average ODI (%)** | **Final Follow-up Average ODI (%)** | **Mean ODI Change** | **P-Value** |
| --- | --- | --- | --- | --- | --- | --- | --- | --- | --- | --- | --- |
| Wentao et al., 2015^9^ | (TDH) Thoracic Disc Herniation | Minimally-Invasive Bilateral Transforaminal Thoracolumbar | Laminectomy, Discectomy and Interbody Fusion | Minimally-Invasive Surgery (bilateral transformational thoracolumbar interbody fusion (bilateral TTIF)) | Back pain (100%); Radicular pain (76%) | 17 | 13.4 | 42.82 | 11.3 | 31.52 | *p* < 0.01 |
| Lin et al., 2021^10^ | (TDH) Thoracic Disc Herniation | Thoracic Transforaminal Percutaneous Endoscopy | Discectomy | Endoscopic thoracic discectomy | Back pain; Radicular | 10 | 12 | 55 | 13 | 42 | *p* < 0.05 |
| Siller et al., 2020^11^ | Degenerative thoracic spinal stenosis | Unilateral Posterior/Bilateral Dorsal | Hemi/Laminectomy | Posterior decompression (unilateral approach) | Pain (26%) | 19 | 35.1 | 40.2 | 19.3 | 20.9 | *p* = 0.003 |
|  | Degenerative thoracic spinal stenosis | Bilateral | Hemi/Laminectomy | Posterior decompression (bilateral approach) | Pain (11%) | 9 | 35.1 | 54.5 | 15.3 | 39.2 | *p* = 0.003 |
| Nacar et al., 2013^15^ | (TDH) Thoracic Disc Herniation | Minimally-Invasive Lateral Transthoracic Trans/Retropleural | Discectomy and Interbody Fusion | Minimally invasive lateral transthoracic trans/retropleural approach | Axial back pain (94%); Radiculopathy (94%); Leg pain (70%) | 33 | 18.2 | 42.4 | 33.2 | 9.2 | *p* < 0.05 |
| Li et al., 2013^8^ | (OLF) Ossification of the Thoracic Ligamentum Flavum | Posterior | Laminectomy | Posterior thoracic extensive decompressive laminectomy | Back pain (42%); Leg pain (11%) | 19 | 53.2 | 66.36 | 25.45 | 40.91 | *p* < 0.05 |
| Li et al., 2021^12^ | (OLF) Ossification of the Thoracic Ligamentum Flavum | Ultrasonic-Assisted Full-Endoscopy | En Bloc Resection | Ultrasonic osteotome assisted full-endoscopic en block resection | Back pain (100%) | 15 | 24 | 53.73 | 21.6 | 32.13 | *p* < 0.05 |
|  | (OLF) Ossification of the Thoracic Ligamentum Flavum | Ultrasonic-Assisted Full-Endoscopy | En Bloc Resection | Ultrasonic osteotome assisted full-endoscopic en block resection | Radicular (100%) |  | 24 |  |  | – |  |
| Wu et al., 2021^13^ | (OLF) Ossification of the Thoracic Ligamentum Flavum | Uniportal Endoscopy | Laminotomy, Bilateral Decompression using One Block Resection | Uniportal Thoracic Endoscopic Laminotomy With Bilateral Decompression Using One Block Resection Technique (TE-ULBD) | Back pain | 28 | 10 | 62 | 21.2 | 40.8 | *p* < 0.05 |
| Hwang et al., 2022^14^ | (OLF) Ossification of the Thoracic Ligamentum Flavum |  | Laminotomy without fusion | Thoracic decompression without instrumented fusion (D) | Back pain | 17 | 24 | 20.6 | 11.1 | 9.5 | *p* < 0.01 |
|  | (OLF) Ossification of the Thoracic Ligamentum Flavum |  | Laminotomy with fusion | Thoracic decompression with instrumented fusion (F) | Back pain | 11 | 24 | 24.4 | 14.5 | 9.9 | *p* < 0.01 |
| Ito Y et al., 2024^39^ | Mixed (thoracic spondylotic myelopathy, OPLL, OLTF, and disk herniation) |  | Decompression with or without fusion |  | Low back pain | 90 | 12 | 45.4 | 30.2 | 15.2 | *p* < 0.001 |
|  |  |  |  |  | Leg pain |  | 12 |  |  | **–** |  |
|  |  |  |  |  | Plantar pain |  | 12 |  |  | **–** |  |
| Baram et al., 2025^19^ | (TDH) Thoracic Disc Herniation |  | Discectomy +/- laminectomy +/- fusion |  | Anterior approach | 34 | 70 |  |  | **–** |  |
|  |  |  |  |  | Posterior approach | 20 | 70 |  |  | **–** |  |
|  |  |  |  |  | Overall | 54 | 70 |  |  | **–** |  |
| Xu et al., 2025^40^ | (OPLL) Ossification of the Posterior Longitudinal Ligament |  | Laminectomy + facetectomy + ventral decompression |  | Overall | 6 | 13.5 | 75.67 | 23.14 | 52.53 | *p* < 0.05 |
|  | (TDH) Thoracic Disc Herniation |  | Laminectomy + facetectomy + ventral decompression |  | Overall | 7 | 11.7 | 66.29 | 21.14 | 45.15 | *p* < 0.05 |
| Chen et al., 2024^41^ | Mixed (OPLL, posterior vertebral osteophytes, hyperplasia of the facet joints, or OLTF) |  | Laminectomy with fusion |  | Overall | 38 | 24.5 | 52.18 | 25.13 | 27.05 | NR |
|  | Mixed (OPLL, posterior vertebral osteophytes, hyperplasia of the facet joints, or OLTF) |  | Conservative |  | Overall | 26 | 24.5 | 51.58 | 58.69 | -7.11 | NR |

**Supplementary Table 6a. JBI Critical Appraisal Checklist for Cohort Studies, applied to the six studies that included a concurrent comparator group. Y, item met; N, item not met; U, unclear; NA, not applicable.**

| **Study** | **1** | **2** | **3** | **4** | **5** | **6** | **7** | **8** | **9** | **10** | **11** | **Yes (/11)** | **Risk of bias** |
| --- | --- | --- | --- | --- | --- | --- | --- | --- | --- | --- | --- | --- | --- |
| Zhang B et al., 2022 | Y | Y | Y | Y | Y | Y | Y | Y | U | N | Y | 9 | Low |
| Soda C et al., 2021 | Y | Y | Y | Y | N | Y | Y | Y | U | N | Y | 8 | Moderate |
| Hwang SH et al., 2022 | Y | Y | Y | Y | N | Y | Y | Y | N | N | Y | 8 | Moderate |
| Baram A et al., 2025 | Y | Y | Y | Y | N | Y | Y | Y | U | N | Y | 8 | Moderate |
| Chen G, Shi J, 2024 | Y | Y | Y | Y | N | Y | Y | Y | U | N | Y | 8 | Moderate |
| Ito Y et al., 2024 | Y | U | Y | Y | N | Y | Y | Y | N | N | Y | 7 | Moderate |
| **Total "Yes" (n = 6)** | **6** | **5** | **6** | **6** | **1** | **6** | **6** | **6** | **0** | **0** | **6** | **48** | **1 low, 5 moderate** |

Y = yes; N = no; U = unclear. Items: 1, both groups recruited from the same population; 2, exposures measured similarly in both groups; 3, exposure measured in a valid, reliable way; 4, confounding factors identified; 5, strategies to deal with confounding stated; 6, participants free of the outcome at the start; 7, outcomes measured in a valid, reliable way; 8, follow-up time reported and sufficient; 9, follow-up complete, or loss to follow-up described and explored; 10, strategies to address incomplete follow-up used; 11, statistical analysis appropriate. Risk of bias was categorised as low (9 or more of the 11 items met, ≥80%), moderate (6 to 8, 50-79%) or high (5 or fewer, <50%).

**Supplementary Table 6b. JBI Critical Appraisal Checklist for Case Series, applied to the 22 studies that did not include a concurrent comparator group.**

| **Study** | **1** | **2** | **3** | **4** | **5** | **6** | **7** | **8** | **9** | **10** | **Yes (/10)** | **Risk of bias** |
| --- | --- | --- | --- | --- | --- | --- | --- | --- | --- | --- | --- | --- |
| Li M et al., 2013 | U | Y | Y | U | U | Y | Y | Y | Y | Y | 7 | Moderate |
| Wentao W et al., 2015 | Y | Y | Y | Y | Y | Y | Y | Y | Y | Y | 10 | Low |
| Zhong ZM et al., 2016 | Y | Y | Y | Y | N | Y | Y | Y | Y | Y | 9 | Low |
| Bransford R et al., 2010 | U | Y | Y | Y | Y | Y | Y | N | Y | U | 7 | Moderate |
| Yang JS et al., 2021 | Y | Y | Y | Y | Y | Y | Y | Y | Y | Y | 10 | Low |
| Carr DA et al., 2017 | Y | Y | Y | Y | Y | Y | Y | Y | Y | Y | 10 | Low |
| Deviren V et al., 2011 | Y | Y | Y | Y | Y | Y | Y | Y | Y | Y | 10 | Low |
| Uribe JS et al., 2012 | U | Y | Y | U | U | Y | Y | Y | Y | Y | 7 | Moderate |
| Yen CP, Uribe JS, 2018 | U | Y | Y | Y | Y | Y | Y | Y | Y | U | 8 | Low |
| Lin W et al., 2021 | Y | Y | Y | U | N | Y | Y | Y | Y | Y | 8 | Low |
| Siller S et al., 2020 | Y | Y | Y | Y | Y | Y | Y | Y | Y | Y | 10 | Low |
| Lisitzky IY et al., 2022 | U | Y | Y | U | U | Y | Y | Y | Y | U | 6 | Moderate |
| Nacar OA et al., 2013 | Y | Y | Y | Y | Y | Y | Y | Y | Y | Y | 10 | Low |
| Quint U et al., 2012 | Y | Y | Y | Y | N | Y | Y | Y | Y | Y | 9 | Low |
| Krishnan A et al., 2020 | Y | Y | Y | U | N | Y | Y | Y | Y | Y | 8 | Low |
| Xin Z et al., 2020 | Y | Y | Y | Y | N | Y | Y | Y | Y | N | 8 | Low |
| Li ZZ et al., 2021 | Y | Y | Y | U | Y | Y | Y | Y | Y | N | 8 | Low |
| Deng Y et al., 2022 | Y | Y | Y | U | Y | Y | Y | Y | Y | Y | 9 | Low |
| Wu PH et al., 2021 | Y | Y | Y | U | Y | Y | Y | Y | Y | Y | 9 | Low |
| Kim JY et al., 2025 | Y | Y | Y | U | N | Y | Y | Y | Y | Y | 8 | Low |
| Xu X et al., 2025 | Y | Y | Y | Y | Y | Y | Y | Y | Y | Y | 10 | Low |
| Ito S et al., 2024 | Y | Y | Y | U | N | Y | Y | Y | Y | Y | 8 | Low |
| **Total "Yes" (n = 22)** | **17** | **22** | **22** | **12** | **12** | **22** | **22** | **21** | **22** | **17** | **189** | **18 low, 4 moderate** |

Y = yes; N = no; U = unclear. Items: 1, clear criteria for inclusion; 2, condition measured in a standard, reliable way for all participants; 3, valid methods used for identification of the condition; 4, consecutive inclusion of participants; 5, complete inclusion of participants; 6, clear reporting of participant demographics; 7, clear reporting of clinical information; 8, outcomes or follow-up results clearly reported; 9, clear reporting of presenting site or clinic demographic information; 10, statistical analysis appropriate. Risk of bias was categorised as low (8 or more of the 10 items met, ≥80%), moderate (5 to 7, 50-79%) or high (4 or fewer, <50%).

**Supplementary Table 7. Synthesis Without Meta-analysis (SWiM) reporting checklist.**

| **Item** | **SWiM reporting item** | **How it is addressed in this review** |
| --- | --- | --- |
| 1 | Grouping studies for synthesis: how studies were grouped for each synthesis, and whether the grouping was decided before or after the results were known. | Methods, Data Analysis and Synthesis. The descriptive synthesis covers the cohorts that could not be meta-analysed, together with the comparisons between surgical approaches and between pain domains that could not be pooled. Within it, studies were grouped by aetiology (thoracic disc herniation, ossification of the ligamentum flavum, ossification of the posterior longitudinal ligament, mixed), by surgical approach (discectomy with versus without fusion) and by pain domain (axial versus non-axial). The grouping follows the review question stated in the PROSPERO protocol and was not derived from the results. |
| 2 | Standardised metric and transformation methods used. | Methods, Data Analysis and Synthesis. The standardised metric is the mean change in score from baseline to final follow-up, on the 0 to 10 cm visual analogue scale and on the Oswestry Disability Index expressed as a percentage. It is derived within each study as the mean pre-operative score minus the mean score at final follow-up, and the summary statistics are taken across those study-level values, so the median change is not the difference between the median pre-operative value and the median value at final follow-up. No transformation was applied. One study reported a numeric rating scale, which is noted where its data are used. |
| 3 | Describe the synthesis methods. | Methods, Data Analysis and Synthesis. Synthesis without meta-analysis, applied only where meta-analysis was not possible. For each group the median, interquartile range and range of the reported changes are given, together with the direction of effect expressed as the number of reported cohorts favouring surgery out of the total. The median and interquartile range were chosen because the number of estimates contributing to each group is small and the median is less sensitive to outlying cohorts. No pooled summary effect is calculated in the descriptive synthesis. Random-effects meta-analyses of the change in VAS and of the change in ODI are reported separately, covering the 19 of 34 VAS cohorts and the nine of 10 ODI cohorts in which the standard deviation of the change was reported or could be imputed. |
| 4 | Criteria used to prioritise results for summary and synthesis. | Methods, Data Analysis and Synthesis, and Results. Priority was given to the random-effects meta-analyses of VAS and ODI. The descriptive synthesis of the cohorts that could not be pooled, the surgical subgroup comparison and the axial versus non-axial comparison are reported as secondary, unadjusted and hypothesis-generating. |
| 5 | Investigation of heterogeneity in reported effects. | Methods, Data Analysis and Synthesis. Heterogeneity was examined by inspecting the spread of the reported changes within each group, reported as the interquartile range and range. It was not quantified statistically for these cohorts, as the absence of standard deviations precludes calculation of I²; for the pooled cohorts heterogeneity was quantified by τ², Cochran's Q and I². |
| 6 | Certainty of evidence: describe any methods used to assess certainty or confidence in the body of evidence. | Methods, Risk of Bias and Quality Assessment; Results, Quality Assessments; Discussion, Limitations. Certainty was rated with GRADE for the two pooled outcomes and is reported in Supplementary Table 9. For the cohorts that could not be pooled, certainty is described narratively from the JBI Critical Appraisal Checklist ratings and from the uncontrolled design of the evidence base. |
| 7 | Data presentation methods: describe the graphical and tabular methods used to present the effects. | Results. Figure 4 presents the distribution of the reported mean scores before surgery and at final follow-up, across the cohorts not included in the meta-analysis, as a box-and-whisker plot showing the median, interquartile range and range. Figures 5 and 6 present the distribution of the reported mean changes in the same form. Supplementary Tables 4 and 5 give the full cohort-level data. |
| 8 | Reporting results: how the results were reported for each synthesis. | Results. For each synthesis the number of contributing studies and reported cohorts, the number of patient-observations, the median and interquartile range of the change, the range, and the direction of effect are reported. No p-value is attached to any between-study or between-subgroup comparison. |
| 9 | Limitations of the synthesis: report any limitations, including the risk of bias due to missing results. | Discussion, Limitations. The synthesis rests on uncontrolled before-and-after data and cannot separate the effect of surgery from natural history or expectation effects. The standard deviation of the change was reported in a minority of cohorts, so the meta-analysis covers 19 of the 34 VAS cohorts and relies on imputation in 14 of them. Sixteen of the 97 reports sought for retrieval could not be obtained, raising the possibility of selection bias. Comparisons between techniques are confounded by indication. |

**Supplementary Table 8. Pre-operative, post-operative and change standard deviations used in the VAS and ODI meta-analyses, with the source of each value. Table, figure and page references in the final column are to the source publication.**

| **Outcome** | **Study** | **Aetiology** | **Cohort** | **Pain domain** | **n** | **Pre-operative mean** | **Final follow-up mean** | **Mean change** | **Standard deviation of the change** | **Source and notes** |
| --- | --- | --- | --- | --- | --- | --- | --- | --- | --- | --- |
| VAS | Zhang 2022 | OLF | DISH+ | back | 58 | 3.37 ± 3.74 | 0.91 ± 1.26 | 2.46 | 3.08 (reported) | Table 3. In the meta-analysis. |
| VAS | Zhang 2022 | OLF | DISH- | back | 109 | 3.45 ± 3.32 | 0.65 ± 1.04 | 2.8 | 2.75 (reported) | Table 3. In the meta-analysis. |
| VAS | Zhong 2016 | OLF | whole cohort | unspecified | 22 | 2.82 ± 3.08 | 0.59 ± 1.05 | 2.23 | 2.71 (imputed) | Abstract and Results, p.79: "2.82 ± 3.08 before surgery and 0.59 ± 1.05 at final". In the meta-analysis. |
| VAS | Yang 2021 | OLF | whole cohort | unspecified | 27 | 5.9 ± 0.9 | 1.3 ± 0.4 | 4.6 | 0.78 (imputed) | Table 2: "VAS score 5.9 ± 0.9 2.2 ± 0.7* 1.3 ± 0.4*#". In the meta-analysis; 1.3 is the final follow-up column. |
| VAS | Li M 2013 | OLF | whole cohort | unspecified | 19 | 6.29 ± 1.83 | 2.18 ± 1.9 | 4.11 | 1.87 (imputed) | Results: "6.29 ± 1.83 before the operation ... and 2.18 ± 1.90 at final follow-up". In the meta-analysis. |
| VAS | Xin 2020 | OLF | whole cohort | axial back | 13 | 5.3 ± 1.2 | 0.69 ± 0.75 | 4.61 | 1.05 (imputed) | Results: "improved from 5.3 ± 1.2 preoperatively to 2.1 ± 0.95 postoperatively, and 0.69 ± 0.75 at the last follow-up". In the meta-analysis. |
| VAS | Li ZZ 2021 | OLF | whole cohort | axial back | 15 | 1.4 ± 1.81 | 0.4 ± 0.63 | 1 | 1.59 (imputed) | Table 3. In the meta-analysis (back pain used; radicular row below is the same patients). |
| VAS | Li ZZ 2021 | OLF | whole cohort | radicular | 15 | 2.8 ± 2.43 | 0.27 ± 0.46 | 2.53 | 2.24 (imputed) | Table 3. Same patients as the row above; used only in the second-domain sensitivity analysis. |
| VAS | Deng 2022 | OLF | biportal endoscopic | leg | 14 | 4.5 ± 2 | 0.8 ± 0.8 | 3.7 | 1.74 (imputed) | Table 2 and Results: "4.5 ± 2.0 vs 0.8 ± 0.8; P < 0.001". In the meta-analysis. |
| VAS | Deng 2022 | OLF | open decompression | leg | 45 | 6.6 ± 1.2 | 1.5 ± 1.2 | 5.1 | 1.2 (imputed) | Table 2: "Pre-operation 6.6 ± 1.2 ... 1-year follow-up 1.5 ± 1.2 (P < 0.001)". In the meta-analysis. |
| VAS | Wu 2021 | OLF | whole cohort | unspecified | 28 | 6.2 (SD not reported) | 1.4 (SD not reported) | 4.8 | 0.93 (reported) | Table 2: "Change in Preop VAS – final VAS 4.80 (0.933)". In the meta-analysis; the standard deviation of the change is reported directly, so no imputation is needed. Pre and post are given as mean (range), not mean ± standard deviation. |
| VAS | Hwang 2022 | OLF | fusion (F-group) | axial back | 11 | 6.5 ± 1.4 | 4.2 ± 1.8 | 2.3 | 1.64 (imputed) | Table 3. In the meta-analysis; Table 4 also reports the change from a linear mixed model as -2.4 ± 1.6 at two years, which is not the raw standard deviation of the change, so the imputed value is used here. |
| VAS | Hwang 2022 | OLF | fusion (F-group) | leg | 11 | 6.9 ± 1.6 | 3.5 ± 2.4 | 3.4 | 2.12 (imputed) | Table 3. Same patients as the row above; second-domain sensitivity analysis only. |
| VAS | Hwang 2022 | OLF | no fusion (D-group) | axial back | 17 | 5.2 ± 2.1 | 3.1 ± 2.2 | 2.1 | 2.15 (imputed) | Table 3. In the meta-analysis; Table 4 also reports the change from a linear mixed model as -2.1 ± 2.0 at two years, which is not the raw standard deviation of the change, so the imputed value is used here. |
| VAS | Hwang 2022 | OLF | no fusion (D-group) | leg | 17 | 5.8 ± 2.3 | 4.5 ± 2.1 | 1.3 | 2.21 (imputed) | Table 3. Same patients as the row above; second-domain sensitivity analysis only. |
| VAS | Kim 2025 | OLF | whole cohort | axial back | 34 | 5.8 ± 1.5 | 2.9 ± 1.3 | 2.9 | 1.41 (imputed) | Table 2. In the meta-analysis. |
| VAS | Kim 2025 | OLF | whole cohort | leg | 34 | 7.4 ± 1.2 | 2.4 ± 0.9 | 5 | 1.08 (imputed) | Table 2. Same patients as the row above; second-domain sensitivity analysis only. |
| VAS | Ito S 2024 | OPLL | whole cohort | axial back (NRS) | 51 | 5.4 ± 3.4 | 3.5 ± 2.4 | 1.9 | 3.03 (imputed) | Table II. In the meta-analysis; NRS rather than VAS. |
| VAS | Ito S 2024 | OPLL | whole cohort | leg (NRS) | 51 | 4 ± 3.4 | 3 ± 2.8 | 1 | 3.14 (imputed) | Table II. Same patients as the row above; second-domain sensitivity analysis only. |
| VAS | Wentao 2015 | TDH | whole cohort | axial back | 17 | 6.65 ± 1.93 | 2.23 ± 0.56 | 4.42 | 1.72 (imputed) | Table 2: "VAS, mean ± SD 6.65 ± 1.93 2.23 ± 0.56*". In the meta-analysis. |
| VAS | Bransford 2010 | TDH | whole cohort | unspecified | 14 | 5.9 (SD not reported) | 2.1 (SD not reported) | 3.8 | Not estimable | Results and Table 1: "improved from an average of 59 to 21" on a 0–100 scale. No dispersion reported; not in the meta-analysis. |
| VAS | Carr 2017 | TDH | whole cohort | unspecified | 51 | 8.31 (SD not reported) | 4.05 (SD not reported) | 4.26 | Not estimable | Results: "improved from 8.31 to 4.05 (P < .01)". No dispersion reported; not in the meta-analysis. |
| VAS | Deviren 2011 | TDH | whole cohort | unspecified | 12 | 9 (SD not reported) | 2.9 (SD not reported) | 6.1 | Not estimable | Results: "9 (range, 7 to 10) ... maintained at 2.9 (range, 0 to ...)". Range only; not in the meta-analysis. |
| VAS | Uribe 2012 | TDH | whole cohort | unspecified | 60 | 7.8 (SD not reported) | 3.1 (SD not reported) | 4.7 | Not estimable | Results: "improved 60% (7.8 to 3.1) from preoperative". No dispersion reported; not in the meta-analysis. |
| VAS | Yen 2018 | TDH | axial pain subgroup | axial back | 5 | 8.2 (SD not reported) | 3.9 (SD not reported) | 4.3 | Not estimable | Results: "VAS score reduced from 8.2 to 3.9". No dispersion reported; not in the meta-analysis. |
| VAS | Yen 2018 | TDH | radicular pain subgroup | radicular | 7 | 7.6 (SD not reported) | 2.1 (SD not reported) | 5.5 | Not estimable | Results: "mean VAS score reduced from 7.6 to 2.1". No dispersion reported; not in the meta-analysis. |
| VAS | Lin 2021 | TDH | whole cohort | unspecified | 10 | 6.1 ± 1.37 | 1.8 ± 0.79 | 4.3 | 1.19 (imputed) | Results: "(6.10 ± 1.37 vs 1.80 ± 0.79, P < 0.05)". In the meta-analysis. |
| VAS | Lisitzky 2022 | TDH | whole cohort | unspecified | 16 | 4.81 ± 1.76 | 2.12 ± 0.89 | 2.69 | 2.09 (calculated) | Table 2, patient-level scores for all 16 patients. In the meta-analysis; means and standard deviations calculated from the patient-level table, so the standard deviation of the change is exact rather than imputed. |
| VAS | Nacar 2013 | TDH | whole cohort | unspecified | 33 | 7.5 (SD not reported) | 3.5 (SD not reported) | 4 | Not estimable | Results: "VAS was 7.5 (range, 3 to 10), which improved to 3.5". Range only; not in the meta-analysis. |
| VAS | Quint 2012 | TDH | whole cohort | unspecified | 167 | 7.14 (SD not reported) | 2.7 (SD not reported) | 4.44 | Not estimable | Results: "7.14, improving to 2.45 points at 6 months, 2.33 at 1 year, increasing to 2.70 at 2 years". No dispersion reported; not in the meta-analysis. 2.70 is the final follow-up value, so the existing extraction is correct. |
| VAS | Krishnan 2020 | TDH | whole cohort | axial back | 18 | 6.94 ± 1.11 | 1 ± 0.9 | 5.94 | 1.02 (imputed) | Results: "The VAS for back improved from 6.94 (1.11) preoperative to 1.0 (0.9) postoperatively". In the meta-analysis; dispersion given in parentheses. |
| VAS | Soda 2021 | TDH | trans-thoracic | radicular | 49 | 4.7 (SD not reported) | 3.2 (SD not reported) | 1.5 | Not estimable | Results and Table 7; VAS reported in bands (0–3, 4–6, 7–10). No dispersion reported; not in the meta-analysis. |
| VAS | Soda 2021 | TDH | retropleural | radicular | 38 | 5 (SD not reported) | 1.39 (SD not reported) | 3.61 | Not estimable | Results and Table 7; VAS reported in bands (0–3, 4–6, 7–10). No dispersion reported; not in the meta-analysis. |
| VAS | Baram 2025 | TDH | whole cohort | unspecified | 54 | 3.7 ± 3.2 | 0.9 ± 1.3 | 2.8 | 3 (reported) | Table 2. In the meta-analysis. |
| ODI | Wentao 2015 | TDH | whole cohort | – | 17 | 42.82 ± 5.08 | 11.3 ± 2.3 | 31.52 | 4.41 (imputed) | Table 2: "ODI, mean ± SD 42.82 ± 5.08 11.3 ± 2.3*". In the meta-analysis. |
| ODI | Lin 2021 | TDH | whole cohort | – | 10 | 55 ± 9.2 | 13 ± 2.36 | 42 | 8.28 (imputed) | Abstract and Results: "13% ± 2.36% vs 55% ± 9.20%, P < 0.05". In the meta-analysis. |
| ODI | Siller 2020 | DTSS | unilateral approach | – | 19 | 40.2 ± 13 | 19.3 ± 12.9 | 20.9 | 12.95 (imputed) | Table: "Mean ODI (range) 40.2 ± 13.0 19.3 ± 12.9 0.003". In the meta-analysis. |
| ODI | Siller 2020 | DTSS | bilateral approach | – | 9 | 54.5 ± 23.6 | 15.3 ± 2.2 | 39.2 | 22.58 (imputed) | Table: "54.5 ± 23.6 15.3 ± 2.2 0.003". In the meta-analysis. |
| ODI | Nacar 2013 | TDH | whole cohort | – | 33 | 42.4 (SD not reported) | 33.2 (SD not reported) | 9.2 | Not estimable | Results: "average ODI score was 42.4 (range, 18 to 70), which improved to 33.2 (range, 0 to 66)". Range only; not in the meta-analysis. |
| ODI | Li M 2013 | OLF | whole cohort | – | 19 | 66.36 ± 10.91 | 25.45 ± 12.19 | 40.91 | 11.6 (imputed) | Results: "decreased from 66.36% ± 10.91% before surgery to 25.45% ± 12.19% after surgery". In the meta-analysis. |
| ODI | Li ZZ 2021 | OLF | whole cohort | – | 15 | 53.73 ± 12.23 | 21.6 ± 6.09 | 32.13 | 10.59 (imputed) | Table 3. In the meta-analysis. |
| ODI | Wu 2021 | OLF | whole cohort | – | 28 | 62 (SD not reported) | 21.2 (SD not reported) | 40.8 | 8.64 (reported) | Results: "significant improvement in the mean ± standard deviation as compared to preoperative ODI in ... final follow up (40.8 ± 8.64)". In the meta-analysis; the standard deviation of the change is reported directly. Pre and post are given as mean (range). |
| ODI | Hwang 2022 | OLF | no fusion (D-group) | – | 17 | 20.6 ± 9.7 | 11.1 ± 5.5 | 9.5 | 8.43 (imputed) | Table 3 (K-ODI). In the meta-analysis; Table 4 also reports the change from a linear mixed model as -9.5 ± 7.9 at two years, which is not the raw standard deviation of the change, so the imputed value is used here. |
| ODI | Hwang 2022 | OLF | fusion (F-group) | – | 11 | 24.4 ± 7.1 | 14.5 ± 6.1 | 9.9 | 6.66 (imputed) | Table 3 (K-ODI). In the meta-analysis; Table 4 also reports the change from a linear mixed model as -9.9 ± 6.2 at two years, which is not the raw standard deviation of the change, so the imputed value is used here. |

**Supplementary Table 9.** GRADE assessment of the certainty of the evidence for the two pooled outcomes. Certainty begins at low for a body of evidence made up of observational studies and is rated down for serious concerns in any domain; it cannot fall below very low.

| **Domain** | **Reduction in VAS pain** | **Reduction in ODI** |
| --- | --- | --- |
| Studies and cohorts | 16 studies, 19 cohorts, 578 patients | 7 studies, 9 cohorts, 145 patients |
| Study design | Uncontrolled observational studies | Uncontrolled observational studies |
| Starting certainty | Low | Low |
| Risk of bias | Serious. No study used blinded outcome assessment or adjusted for confounding; 22 of the 28 included studies had no comparator group, and seven restricted eligibility to patients with complete records or a minimum duration of follow-up. | Serious. The same limitations apply; every contributing study was uncontrolled or unblinded. |
| Inconsistency | Serious. I² = 94.5%, τ² = 1.49, and the 95% prediction interval runs from 0.80 to 6.10 cm. | Very serious. I² = 97.3%, τ² = 158.3, and the 95% prediction interval runs from −2.1 to 60.8 points, so it includes no change. |
| Indirectness | Not serious. The population, intervention and outcome are those of the review question. | Not serious, although the ODI measures pain-related disability rather than pain intensity. |
| Imprecision | Not serious. 578 patients; the 95% confidence interval, 2.88 to 4.03 cm, lies entirely above the minimum clinically important difference of 1.5 to 2.0 cm. | Not serious. The 95% confidence interval, 20.9 to 37.8 points, lies entirely above the minimum clinically important difference of 10 to 12%. |
| Publication bias | Undetected. Egger's test was significant, but the asymmetry runs opposite to the pattern expected of publication bias. | Undetected. Egger's test gave no evidence of asymmetry. |
| Pooled effect | Mean reduction 3.45 cm (95% CI 2.88-4.03) | Mean reduction 29.4 points (95% CI 20.9-37.8) |
| Certainty of the evidence | Very low | Very low |
